# Retinal pathology in experimental optic neuritis is characterized by retrograde degeneration and gliosis

**DOI:** 10.1186/s40478-019-0768-5

**Published:** 2019-07-17

**Authors:** Praveena Manogaran, Marijana Samardzija, Anaïs Nura Schad, Carla Andrea Wicki, Christine Walker-Egger, Markus Rudin, Christian Grimm, Sven Schippling

**Affiliations:** 1 Department of Information Technology and Electrical Engineering, Swiss Federal Institute of Technology, Zurich, Switzerland; 2 Neuroimmunology and Multiple Sclerosis Research, Clinic for Neurology, University Hospital Zurich and University of Zurich, Zurich, Switzerland; 3 Department of Ophthalmology, Lab for Retinal Cell Biology, University of Zurich, Zurich, Switzerland; 4 Department of Biology, University of Zurich, Zurich, Switzerland; 5 Department of Health Sciences and Technology, Swiss Federal Institute of Technology, Zurich, Switzerland; 6 Institue for Biomedical Engineering, Swiss Federal Institute of Technology and University of Zurich, Zurich, Switzerland; 7 Institute of Pharmacology and Toxicology, University of Zurich, Zurich, Switzerland

**Keywords:** Optical coherence tomography, Optic neuritis, Retina, Experimental autoimmune encephalomyelitis, Neuro-axonal degeneration, Gliosis

## Abstract

The exact mechanisms and temporal sequence of neurodegeneration in multiple sclerosis are still unresolved. The visual pathway including its unmyelinated retinal axons, can serve as a prototypic model of neurodegeneration in experimental optic neuritis. We conducted a longitudinal study combining retinal imaging through optical coherence tomography (OCT) with immunohistochemical analyses of retinal and optic nerve tissue at various time points in experimental autoimmune encephalomyelitis (EAE).

Inner retinal layer (IRL) thickness was measured in 30 EAE and 14 healthy control C57BL/6 J mice using OCT. Distribution of marker proteins was assessed by immunofluorescence staining and retinal mRNA levels were assayed using real-time PCR. Histological morphology was evaluated on light and electron microscopy images.

Signs of inflammatory edema 11 days post immunisation coincided with IRL thickening, while neuro-axonal degeneration throughout the disease course contributed to IRL thinning observed after 20 days post immunisation. Retinal pathology, including axonal transport impairment, was observed early, prior to cellular infiltration (i.e. T-cells) in the optic nerve 11 days post immunisation. Yet, the effects of early retinal damage on OCT-derived readouts were outweighed by the initial inflammatory edema. Early microglial activation and astrocytosis was detected in the retina prior to retinal ganglion cell loss and persisted until 33 days post immunisation. Müller cell reactivity (i.e. aquaporin-4 and glutamine synthetase decrease) presented after 11 days post immunisation in the IRL. Severe neuro-axonal degeneration was observed in the optic nerve and retina until 33 days post immunisation.

Initial signs of retinal pathology subsequent to early glial activity, suggests a need for prophylactic treatment of optic neuritis. Following early inflammation, Müller cells possibly respond to retinal pathology with compensatory mechanisms. Although the majority of the IRL damage observed is likely due to retrograde degeneration following optic neuritis, initial pathology, possibly due to gliosis, may contribute further to IRL thinning. These results add morphological substrate to our OCT findings. The extent and rapid onset of axonal and neuronal damage in this model appears relevant for testing interventions scaled to human optic neuritis.

**Electronic supplementary material:**

The online version of this article (10.1186/s40478-019-0768-5) contains supplementary material, which is available to authorized users.

## Introduction

Neurodegeneration is a key factor for irreversible disability observed in multiple sclerosis (MS) [1]. Although previously thought to be a consequence of inflammatory mediated myelin loss, neurodegeneration independent of demyelination has been observed in both murine models of MS [2, 3] and human post-mortem retinal tissue [4]. Neurodegeneration in the afferent visual pathway is prevalent in MS, most frequently following optic neuritis (ON; an inflammation of the optic nerve) and often resulting in functional visual impairment that can persist even after treatment [5, 6]. Damage from the optic nerve can propagate into the retina, by processes of retrograde degeneration, contributing to visual impairment observed in MS [7].

Similar to findings in human studies, alterations in the retina [8, 9], optic nerve [10–13], tract [11, 12], chiasm [14], and radiations [15] have been reported in mouse models of MS. Approximately 70–92% of eyes develop ON 11 days post immunisation (dpi) in experimental ON mouse models, making it ideal for investigating visual pathway dysfunction [11, 16, 17]. Although numerous target autoantigens exist for murine models of MS, myelin oligodendrocyte glycoprotein (MOG) induced experimental autoimmune encephalomyelitis (EAE) appears to have predilections for optic tract pathology and often results in the development of bilateral ON [18]. For example, MOG-specific T-cells induce a greater density of lesions in the optic nerve compared to other models such as myelin basic protein (MBP) induced EAE [18]. Moreover, murine and human retinas share many commonalities including comparable retinal layers with the primary difference being generally thinner layers and the lack of a macula in mice [19].

Optical coherence tomography (OCT) is a rapidly evolving, non-invasive imaging technique that has become a prominent tool in MS research. OCT not only assesses structural injury in the visual system, frequently affected in MS, but can also be utilized as a powerful marker for more global central nervous system (CNS) neurodegeneration [20]. The inner retinal layer (IRL) includes the unmyelinated axons, cell bodies, and dendrites of the third level CNS retinal neurons, retinal ganglion cells (RGC), which can be quantified using OCT. This is ideal for assessing neuro-axonal degeneration independent of demyelination and might constitute a beneficial model for testing treatments targeting neurodegeneration specifically. Furthermore, numerous studies have found significant associations between OCT measures of retinal atrophy and magnetic resonance imaging detected brain tissue loss both globally, [21–23] and within the visual pathway [24–27], further solidifying the potential of OCT for the assessment of CNS neurodegeneration in MS.

Several studies have assessed retinal pathology using OCT in experimental models of ON [3, 11, 16, 28–32]. In particular, studies have revealed initial phases of IRL thickening [11, 31] around the optic nerve head (ONH) followed by severe thinning at later stages of the disease in EAE mice compared to healthy controls [16]. This resembles what has been observed in some cases of MS-related acute ON, with an initial phase of swelling in the peripapillary retinal nerve fiber layer (pRNFL) that diminishes over time, revealing severe degeneration or thinning [33, 34].

Regardless of the prominent use of OCT in MS and ON research, it remains uncertain what the changes in IRL thickness indicate in the context of pathological processes. Few studies have attempted to associate OCT derived retinal layer findings with anatomical correlates [35, 36], however, the topic has only been marginally explored and it is still not clear how MS pathology might affect the inner retina. Currently, it is assumed that increases in pRNFL thickness in human acute-ON or IRL thickness in EAE are due to edema related to the inflammatory autoimmune response on the myelinated portion of the optic nerve in early stages, whereas, IRL thinning observed later in the disease is thought to reflect axonal loss, neurodegeneration, or retrograde degeneration following ON. However, these speculations are based primarily on correlation studies in humans [25, 37, 38]. Furthermore, thickness changes in the inner nuclear layer (INL) and outer plexiform layer (OPL) are relatively unexplored in mouse models of MS, including how these changes might relate to Müller cell reactivity.

Therefore, the objective of the current study was to add structural meaning to OCT findings using immunohistochemical analyses of tissue specimen and to explore the biological mechanisms of visual pathway damage in experimental ON longitudinally.

## Materials & methods

### Mice and EAE induction

Studies were performed in conformity with the Swiss Animal Welfare Law (License No.: 078/15). A total of 44 female 16–18 weeks old C57BL/6JRj (Janvier, Le Genest-St Isle, France) [39, 40] mice were examined in this study. EAE was induced in 30 mice by immunisation with an emulsion of MOG_35–55_ (Hooke Laboratories, Lawrence, MA, USA), in complete Freund’s adjuvant (CFA), injected subcutaneously into both flanks (0.2 mg/mouse), followed by intraperitoneal administration of pertussis toxin in phosphate-buffered saline (PBS) on day 0 of immunisation and again on day 1 (4 μg/mL/mouse/day). Animals were weighed and observed/scored daily after showing the first signs of symptoms, as assessed by the EAE disability score (0 = no detectable signs of EAE, 0.5 = distal limp tail, 1 = complete limp tail, 1.5 = limp tail and hind limp weakness, 2.0 = unilateral partial hind limb paralysis, 2.5 = bilateral partial hind limb paralysis, 3.0 = complete bilateral hind limb paralysis, 3.5 = complete bilateral hind limb paralysis and partial forelimb paralysis, 4.0 = moribund, 5.0 = death). All mice had access to food and water ad libitum and were housed in a light-dark cycle of 12:12 h. Directly after EAE induction, food and water was placed on the cage floor to facilitate food and water intake of disabled animals. To reduce artificial light related damage to the eyes, all cages were kept on the same rack/level with the same distance from light sources (furthest away) and provided red houses.

### OCT assessment

The OCT methodology is reported in line with the APOSTEL recommendations [41]. A Spectralis® OCT-2 Plus device (Heidelberg Engineering, Heidelberg, Germany) was utilized for the OCT assessment and operated by a single user (P.M.). The device is equipped with a TruTrack® eye tracking program that ensures exact repositioning of the laser beam and optimizes reliability of repeated, longitudinal measurements. To adapt the system for mice imaging, a commercially available lens (25 diopter) was fitted to the system. Anesthesia was induced with 3.0% isoflurane (Piramal Healthcare Limited, India) in a 1:2 O_2_/air mixture. Anesthesia was then reduced to 2.0% isoflurane in a 1:2 O_2_/air mixture for the remainder of the examination while the animals were spontaneously breathing. The pupils of both eyes were dilated (Mydriaticum Dispersa; Tropicamidum 5 mg/ml, OmniVision, Neuhausen, Switzerland) prior to the examination. Animals were placed on a custom-built positioning device with an integrated water heating system to avoid hypothermia. Ocular lubrication was maintained using a gel (Lacrinorm; Carbomerum 980 2 mg/ml, Bausch & Lomb Swiss AG, Zug, Switzerland) and a vendor supplied atraumatic focal contact lens was placed over the eye to improve image acquisition quality.

Horizontal and vertical line scans were used for positioning the scan protocol over the ONH in the center of the fundus image. OCT measurements were obtained under ambient light conditions with a focus distance of 40 diopters. A high-resolution volume scan was performed over the ONH involving 25 consecutive horizontal B-scans (148 μm between B-scans; ART 50; 512 A-scans each; 3.5 × 3.5 mm). A built-in semi-automated segmentation tool (Spectralis software version 6.7.13.0, Eye Explorer software version 1.9.14.0) followed by manual correction from qualified users (P.M., A.N.S.) was used to obtain the IRL, INL, and OPL thicknesses. A 1.0, 2.22, 3.45 mm circular grid was placed over the ONH and the average thickness of all the sections, excluding the center (due to excessive noise), was obtained for the OCT measurements. Both eyes were examined and each scan was assessed for sufficient signal strength, adequate illumination, and accurate beam placement. All scans had a quality of at least 20 dB. OCT examinations occurred on the same day or days prior to sacrificing the mice for ex-vivo analysis. Each mouse received OCT examinations longitudinally at four out of the seven different following time points: baseline (prior to EAE induction; *n* = 11), 7 days post immunisation (dpi; EAE: *n* = 5, control: *n* = 3), 9 dpi (EAE: *n* = 5, control: *n* = 3), 11 dpi (EAE: *n* = 5, control: *n* = 2), 15 dpi (EAE: *n* = 3), 20 dpi (EAE: *n* = 4, control: *n* = 3), and 28 dpi (EAE: *n* = 7, control: *n* = 2).

### Immunofluorescence

Optic nerves and eyes were extracted and fixed for 4 h in 4% paraformaldehyde (in PBS) at 4 °C. The eyes were separated from the optic nerve and pre-embedded in 2% agarose gel for better positioning. The eyes and optic nerves were dehydrated using the LOGOS J Microwave Hybrid Tissue Processor (Milestone SRL, Bergamo, Italy) and then embedded into paraffin blocks. Five micron thick retinal sections were cut dorso-ventrally through the ONH (including a small segment of the optic nerve) and 5 μm thick longitudinal optic nerve sections were cut using a Microm HM 355S Rotary Microtome with a STS Section-Transfer-System (Thermo Fisher Scientific, Waltham, MS, USA).

For immunostaining, sections were heated to 60 °C for 1 h, washed several times in xylene, followed by rehydration with a series of decreasing ethanol concentrations. For some primary antibodies (see Table 1), unmasking was performed with sodium citrate buffer pH 6.0 (10 mM sodium citrate, 0.05% Tween 20) at 90 °C for 20 min, followed by 2 washes for 5 min with distilled water and then one wash for 5 min in PBS. All sections were incubated with blocking solution (3% normal goat serum in PBS/ 0.3% Triton X-100 or 2% horse serum in PBS/ 0.2% TritonX-100) for 1 h at room temperature. Primary antibodies (Table 1) were applied in blocking solution overnight at 4 °C. Slides were washed 3 times with PBS and incubated with anti-mouse or anti-rabbit Alexa Fluor 568 or 488 (Thermo Fisher Scientific, Waltham, MA, USA; 1:500) or anti-goat Cy3-conjugated secondary antibodies (Jackson Immuno Research, Soham, UK; 1:500) in blocking solution for 1 h at room temperature. Slides were then washed two times with PBS, and nuclei were counter stained with 4′, 6-diamidino-2-phenylindole (DAPI) in PBS for 6 min at room temperature. Following the last wash, slides were mounted and analyzed using a fluorescent microscope (Zeiss, Axioplan Feldbach, Switzerland) and a confocal microscope (Leica TCS SP8, Leica Application Suite X, V 3.1.1.15751, oil immersion at 63x magnification).

**Table 1:** Primary antibodies used for immunofluorescence

Antigen	Host	Dil.	HIER	Catalog No.	Company
Glial fibrillary acidic protein (GFAP)	Mouse	1:500	Yes	G-3893	Sigma, St Louis, USA
Allograft inflammatory factor 1 (IBA1)	Rabbit	1:250	Yes	019–19741	Wako, Neuss, Germany
Neurofilament-M (NEFM)	Mouse	1:500	No	RMO-270	Thermo Fisher Scientific, Waltham, USA
CD3	Rabbit	1:100	Yes	NB600–1441	Novus Biologicals, Littleton, USA
Albumin (ALB)	Rabbit	1:500	No	RARaAlb	Nordic Immunology, Tilburg, Netherlands
Myelin basic protein (MBP)	Rabbit	1:1000	No	ab216668	Abcam, Cambridge, UK
Neuronal nuclei (NEUN)	Mouse	1:250	No	MAB377	Merck Millipore, Billerica, USA
Glutamine synthetase (GS)	Mouse	1:250	Yes	MAB302	Merck Millipore, Billerica, USA
Alzheimer precursor protein (APP)	Mouse	1:100	Yes	MAB348	Merck Millipore, Billerica, USA
Aquaporin-4 (AQP4)	Goat	1:250	No	sc-9888	Santa Cruz Biotechnology, USA
Transmembrane protein 119 (TMEM119)	Rabbit	1:200	Yes	G-9264	Sigma, St Louis, USA
CD68	Mouse	1:100	Yes	MAB1435	Merck Millipore, Billerica, USA

*HIER* heat-induced epitope retrieval, *Dil.* Dilution

Neuronal nuclei (NEUN) and DAPI stained retinal sections imaged with the fluorescent microscope were stitched into panoramic retinal images in Adobe Photoshop CS6 Extended (Adobe Systems, Inc., San Jose, CA, USA) using the Photomerge function without either blending or geometric distortion corrections. Counting of positively labeled NEUN cells co-stained with DAPI was performed by two investigators (P.M., A.N.S.) blinded to subject information, including group assignment of each animal. An average of the two investigators NEUN positive cell count was used for the statistical analysis. For immunofluorescence, mice were sacrificed 7 dpi (EAE: *n* = 3, control: *n* = 1), 9 dpi (EAE: *n* = 3, control: *n* = 1), 11 dpi (EAE: *n* = 3, control: *n* = 1), 15 dpi (EAE: *n* = 3, control: *n* = 1), 20 dpi (EAE: *n* = 3, control: *n* = 1), 28 dpi (EAE: *n* = 3, control: *n* = 2), and 33 dpi (EAE: *n* = 2, control: *n* = 2).

### TUNEL assay

Terminal deoxynucleotidyl transferase mediated dUTP nick-end labelling (TUNEL) was performed according to the manufacturer’s recommendations (Roche Diagnostics, Rotkreuz, Switzerland) on 5 μm paraffin retinal sections to detect RGC death. Immunofluorescent signals were analyzed with a digital microscope (Zeiss, Axioplan Feldbach, Switzerland) and all RGCs positive for TUNEL were counted at each time point. For the TUNEL staining, retinal sections were obtained from 2 healthy controls and from EAE mice at 9 dpi (*n* = 1), 11 dpi (*n* = 1), 15 dpi (*n* = 2), 20 dpi (*n* = 2), 28 dpi (*n* = 2), and 33 dpi (*n* = 2).

### RNA isolation and semi-quantitative real-time polymerase chain reaction

Retinas were isolated through a slit in the cornea, frozen in liquid nitrogen and stored at − 80 °C. Total RNA was extracted using an RNA isolation kit (NucleoSpin; Macherey-Nagel) including a DNAse treatment to remove residual genomic DNA. 1 μg of RNA, oligo(dT) and M-MLV reverse transcriptase (Promega, Fitchburg, WI, USA) were used to prepare cDNA. To analyze gene expression by real-time PCR, 10 ng of cDNA template were amplified using a polymerase chain reaction (PCR) polymerase ready mix (PowerUp Syber Green Master Mix, Thermo Fisher Scientific), specific primer pairs (Table 2), and a thermocycler (ABI QuantStudio 3, Thermo Fisher). Expression levels were normalized to β-actin (*Actb*) and relative expression was calculated with the ThermoFisher software using the comparative threshold cycle method (∆∆CT) and control samples for calibration [42]. The healthy controls from all time points were grouped together for analysis. The mice in each group and the time points assessed with real-time PCR were the same as the ones included for the immunofluorescence assessment in Section 2.3. The eyes and optic nerves from each mouse were randomly pre-assigned for either immunofluorescence or real-time PCR analysis.

**Table 2:** Primers used for real-time PCR

Gene	Forward (5′-3′)	Reverse (5′-3′)	Product (bp)
*Actb*	CAACGGCTCCGGCATGTGC	CTCTTGCTCTGGGCCTCG	153
*Pou4f1 (Brn3a)*	CGCCGCTGCAGAGCAACCTCTT	TGGTACGTGGCGTCCGGCTT	130
*Bdnf*	CAAAGCCACAATGTTCCACCAG	GATGTCGTCGTCAGACCTCTCG	213
*Casp1*	GGCAGGAATTCTGGAGCTTCAA	GTCAGTCCTGGAAATGTGCC	138
*Mcp1 (Ccl2)*	GGCTCAGCCAGATGCAGTTA	CTGCTGCTGGTGATCCTCTT	108
*Gfap*	CCACCAAACTGGCTGATGTCTAC	TTCTCTCCAAATCCACACGAGC	240
*Il1b*	ACTACAGGCTCCGAGATGA	CGTTGCTTGGTTCTCCTTG	141
*Tnf*	CCACGCTCTTCTGTCTACTGA	GGCCATAGAACTGATGAGAGG	92
*Aqp4*	TACTGGAGCCAGCATGAATC	CCACATCAGGACAGAAGACA	149
*Rlbp1 (Cralbp)*	CCTTTCCAGTCGGGACAAGTATG	GGGTTTCCTCATTTTCCAGCAG	140

### Histology for light and electron microscopy

To evaluate retinal and optic nerve morphology, eyes and optic nerves were enucleated and fixed in 2.5% glutaraldehyde in cacodylate buffer (pH 7.2, 0.1 M) overnight at 4 °C. For the eyes, the cornea and lens were removed and by cutting through the ONH, the nasal and temporal halves of the eyecups were separated. For the optic nerve, the anterior portion was sectioned for cross-sectional segments and the posterior part was sectioned for longitudinal segments. All the samples were washed in cacodylate buffer twice for 15 min and incubated in 1% osmium tetroxide in cacodylate buffer for 1 h. The samples were then dehydrated in a series of increasing ethanol concentrations and embedded in Epon 812 plastic with propylene oxide. Semi-thin sections (0.5 μm) were counterstained with methylene blue and analyzed by light microscopy (Axioplan; Zeiss, Jena, Germany).

Ultra-thin sections (100 nm) were cut and transferred onto silicon wafers. They were stained with an alcoholic solution of 2% uranyl acetate and Reynolds lead citrate in sodium hydroxide. The sections were scanned using a Zeiss Merlin ultra-high resolution field emission scanning electron microscope (Zeiss, Oberkochen) at 2 kV acceleration voltage and a beam current of 400pA with the Everhart-Thornley-Detector. The mosaic image was acquired via an ATLAS 5 System (Zeiss, Oberkochen). Stitching was done with TrakEM2, a FIJI plugin [43] and was exported as PNG for further analysis. All electron microscopy images have a resolution of 8 nm per pixel. Morphological structure of the optic nerve and retinal sections were assessed qualitatively. For histology, mice were sacrificed 7 dpi (EAE: *n* = 3, control: *n* = 2), 11 dpi (EAE: *n* = 3), and 28 dpi (EAE: *n* = 4, control: *n* = 2).

### Statistical analysis

Statistical analysis was performed using R: a language and environment for statistical computing (R Core Team, 2016, V: 1.1.453; https://www.r-project.org/). For OCT measurements, a linear mixed effects model (function lmer in library lme4, V 1.1–17) was used to account for intra-subject, inter-eye dependencies with group and time points as covariates. The residuals of the model were assessed for normality using Q-Q plots (function qqnorm in default core R library) and histograms (function hist in default core R library) to ensure the model fit the data properly. The least squares mean test (function emmeans in library emmeans, V1.2.4) revealed the differences between specific comparisons and included a Bonferroni correction for multiple comparisons. Based on these models, we assessed the differences between study groups at each time point. For the NEUN cell count, an ANOVA (function lm in default core R library) was used to assess the differences between EAE and healthy controls at each time point. Pairwise comparisons incorporating Bonferroni correction (function emmeans in library emmeans, V1.2.4) were completed to evaluate differences between specific comparisons. For real-time PCR analysis, statistical differences between EAE and healthy controls at different time points was calculated using ANOVA (function lm in default core R library) and corrected for multiple comparisons using Bonferroni-Holm correction (function p.adjust in default core R library). *p*-values equal to or below 0.05 were taken to be statistically significant for all measurements.

## Results

### Clinical assessment

**Fig. 1:**
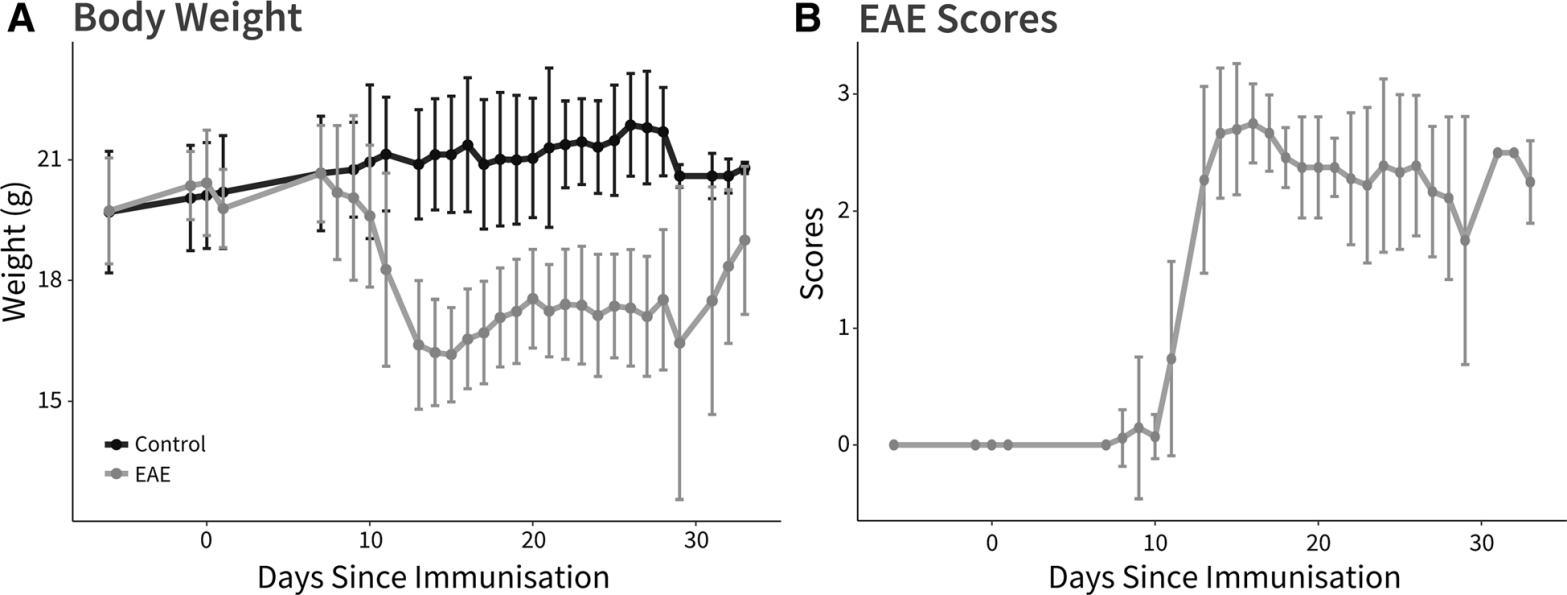
Clinical assessment of healthy and EAE induced C57BL/6 J mice. **a** Body weight over the observational period for both healthy control C57BL/6 J (black) and EAE induced (grey) mice, mean ± SD. **b** EAE clinical scores longitudinally in EAE induced mice, mean ± SD. EAE: experimental autoimmune encephalomyelitis.

Initial body weight loss was observed in EAE mice 10 days after immunisation, with the greatest decline detected at peak of disease (15 dpi), followed by a gradual weight increase until the final observation time point (Fig. 1a). Clinical symptoms began with onset of motor impairment at 11 dpi, peak of disability at 15 dpi, partial recovery of symptoms by 20 dpi, followed by stabilization of symptoms until the final observational time point (Fig. 1b).

### OCT detected retinal changes in EAE and healthy mice

**Fig. 2:**
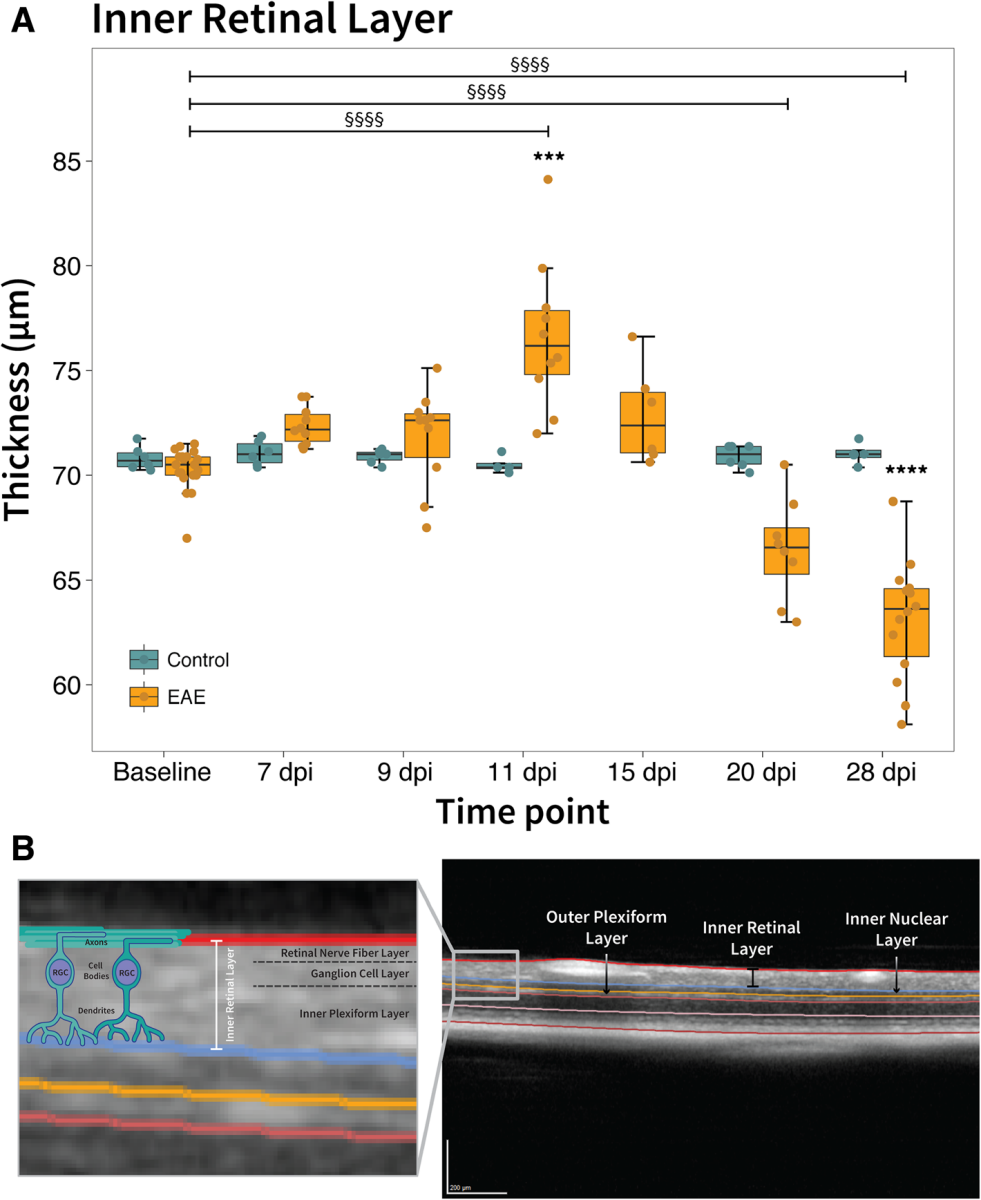
Inner retinal layer thickness increased at onset and decreased at later time points in EAE. **a** Boxplots of the inner retinal layer (IRL) thickness over time in EAE (yellow) and healthy controls (blue). **b** Optical coherence tomography retinal B-scan of healthy C57BL/6 J mouse with segmented layers including the IRL, inner nuclear layer and outer plexiform layer. Inset includes a diagram of the IRL composed of retinal ganglion cells (RGC). The retinal nerve fiber layer consists of the unmyelinated axons of the RGCs, with their cell bodies and dendrites located within the ganglion cell layer and inner plexiform layer respectively. * *p*-values for comparison between EAE and control mice (**** *p* < 0.00001, *** *p* < 0.0001), § *p*-value for comparison between baseline and recovery in EAE mice (§§§§ *p* < 0.00001). EAE: experimental autoimmune encephalomyelitis, dpi: days post immunisation. Scale bars: 200 μm

In our previous study [11], IRL thickness was examined at baseline, 11, 15, and 28 dpi according to expected changes in EAE scores. The current study included additional time points (7, 9, and 20 dpi) to determine when first signs of structural retinal alterations are observed and to further characterize IRL thickness changes over time. IRL thickness in healthy control mice remained constant and within error limits throughout the observational period. Similarly, IRL thickness of EAE mice at baseline (mean ± SD: 70.27 ± 1.13 μm) was not statistically different from healthy controls at baseline (70.81 ± 0.56 μm). First signs of IRL thickening in EAE mice were observed at 11 dpi (76.65 ± 3.55 μm, *p* = 0.001) but not at prior time points, while a statistically significant decrease was detected at 28 dpi (63.14 ± 2.83 μm, *p* = 0.00002) compared to healthy controls at baseline (Fig. 2). In comparison to baseline values in EAE mice, IRL significantly increased in thickness until 11 dpi (*p* < 0.00001), and significantly decreased at 20 dpi (66.47 ± 2.47 μm, *p* = 0.0003) as well as at 28 dpi (*p* < 0.00001; Fig. 2a). However, the outer retinal layers that were not examined in our prior study, exhibited no statistically significant difference in INL or OPL thickness between EAE and healthy control mice (Fig. 2b). The means and SD of all OCT measurements can be found in Table 3.

**Table 3:** Mean values of each optical coherence tomography measurement along with the standard deviation in parentheses for healthy controls and EAE mice at each time point

Time points	IRL (μm)		INL (μm)		OPL (μm)	
	Control	EAE	Control	EAE	Control	EAE
Baseline	70.81 (0.56)	70.27 (1.12)	23.31 (1.93)	22.88 (2.95)	21.02 (0.62)	20.05 (1.17)
7 DPI	71.06 (0.60)	72.36 (0.91)	22.10 (2.39)	22.58 (1.37)	19.75 (0.59)	18.81 (1.54)
9 DPI	70.90 (0.33)	71.83 (2.34)	21.35 (2.17)	22.08 (2.86)	19.31 (0.47)	19.59 (1.39)
11 DPI	70.50 (0.43)	76.65 (3.55)	22.16 (2.65)	21.05 (1.36)	20.94 (0.52)	19.59 (1.21)
15 DPI	NA	72.85 (2.33)	NA	20.83 (1.23)	NA	19.88 (0.77)
20 DPI	70.50 (0.55)	66.47 (2.47)	21.52 (1.87)	21.69 (2.94)	19.63 (0.78)	19.89 (2.20)
28 DPI	71.03 (0.56)	63.14 (2.83)	22.22 (2.40)	22.67 (2.66)	21.06 (0.51)	19.86 (1.66)

*EAE* experimental autoimmune encephalomyelitis, *IRL* inner retinal layer, *INL* inner nuclear layer, *OPL* outer plexiform layer, *DPI* days post immunisation, *NA* not applicable

### Evaluating blood-retinal-barrier (BRB) disruption

To characterize the histopathological basis underlying these OCT findings, extensive ex-vivo analysis was performed at various time points in both the retina and optic nerve. Firstly, signs of BRB disruption were assessed with albumin immunostaining in retinal samples. Diffuse albumin staining was observed outside and around blood vessels in the retinal superior vascular plexus of EAE mice at 9 dpi and 11 dpi, but not at later time points compared to healthy controls (Additional file 1). In healthy controls, albumin was present only within the blood vessels at all time points (Additional file 1).

### Microglial and astroglial response in the retina and optic nerve

**Fig. 3:**
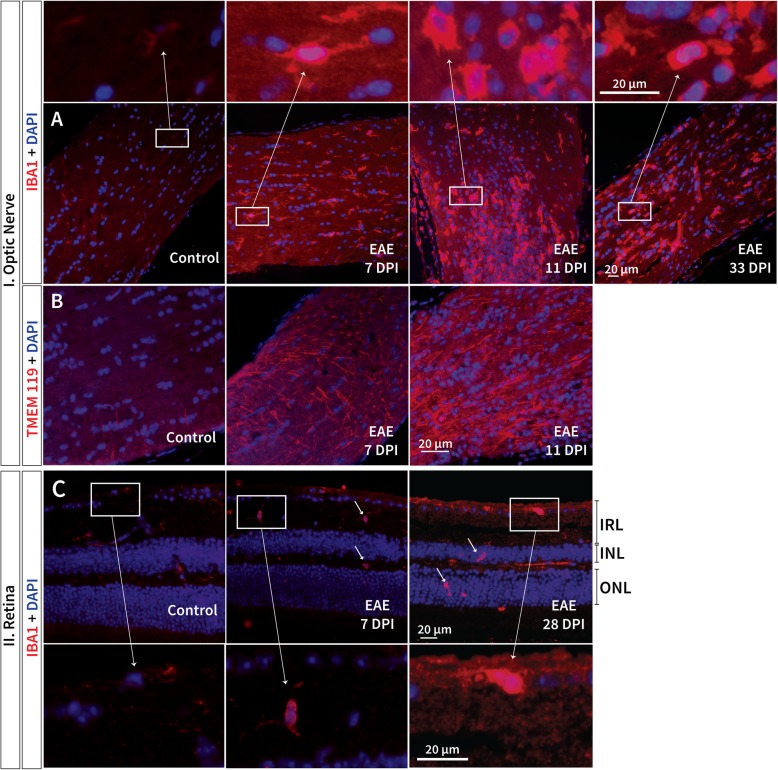
Microgliosis presented early in EAE and persisted until the final observational time point. Increased microglial cell (IBA1) presence was observed as early as 7 days post immunisation (dpi) in both the **a**/**b** optic nerve and **d** retina (arrows), persisting until the final observational time point in EAE mice. **b** TMEM119 marked resident microglial cells observed at 7 dpi and 11 dpi in EAE mice, **a** while in the optic nerve, a significant increase in microglial cells at 11 dpi also coincided with cellular infiltration. The insets **a**/**c** display examples of microglial cells in controls (ramified: weak signal, long branched processes) and EAE mice (amoeboid: strong signal, round)

To delineate the microglial response throughout the disease course, allograft inflammatory factor 1 (IBA1) staining was performed in retinal and optic nerve samples. Increased microglial cell presence was observed as early as 7 dpi in both the retina and optic nerve and persisted until the final observational time point (Fig. 3a and c). The microglial response was more pronounced in the optic nerve compared to the retina (Fig. 3a and c). In the optic nerve, the greatest presentation of microglial cells occurred at 11 dpi, coinciding with extensive cellular infiltration (as illustrated by the increase in the number of nuclei stained with DAPI; Fig. 3a). To assess if the microglial cells observed early on in the disease were resident microglia, optic nerve sections were stained for transmembrane protein 119 (TMEM119; Fig. 3b) [44]. Based on the TMEM119 staining, an increased number of resident microglia was observed in the optic nerve of EAE mice as early as 7 dpi, which intensified further at 11 dpi (Fig. 3b).

**Fig. 4:**
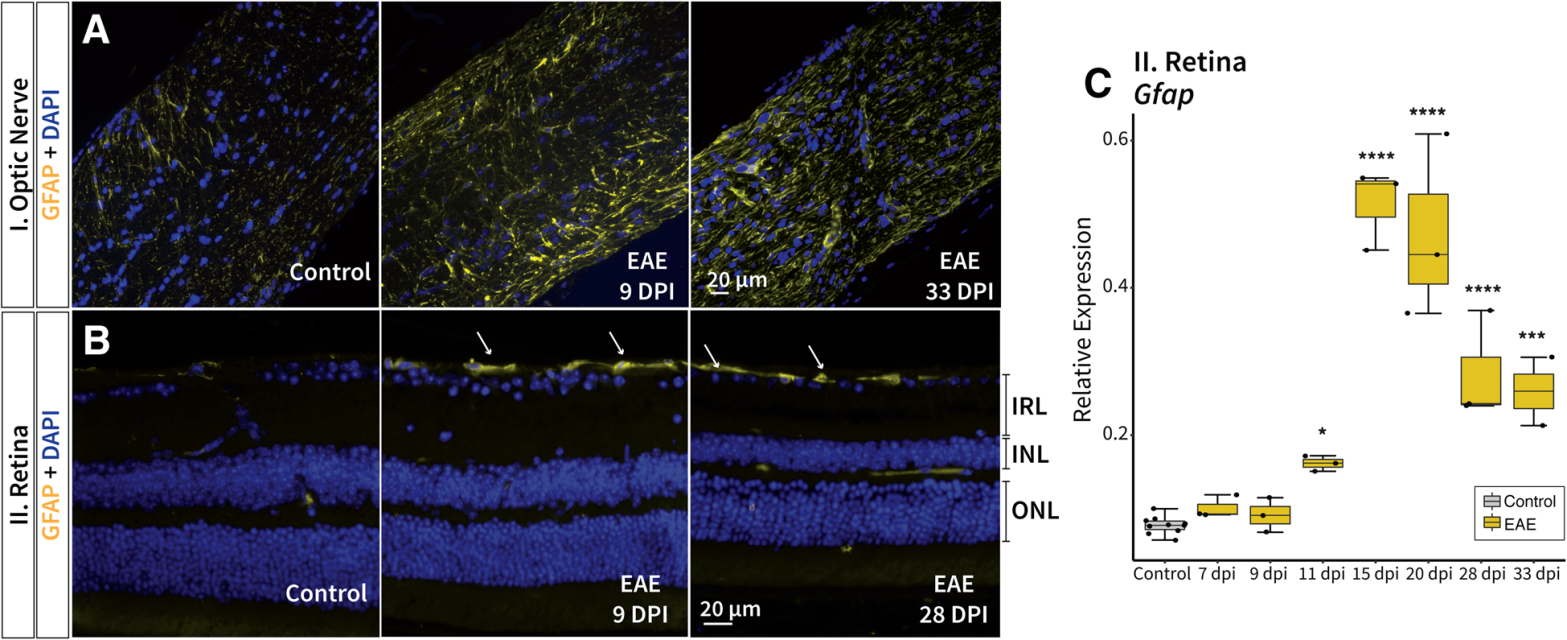
Astrocytosis appeared subsequent to microgliosis in EAE and also persisted until the final time point. Increased GFAP signal (associated with gliosis) presented at 9 dpi and also remained until 33 dpi in both the **a** optic nerve and the **b** retina (arrows). **c** mRNA expression of *Gfap* in EAE retinas significantly increased from 11 dpi to 33 dpi, tapering off at the final two observational time points. *p*-values for comparison between EAE and control mice (**** *p* < 0.00001, *** *p* < 0.0001, * *p* < 0.05). EAE: experimental autoimmune encephalomyelitis, GFAP: glial fibrillary acidic protein, IRL: inner retinal layer, INL: inner nuclear layer, ONL: outer nuclear layer

To assess the astroglial response, glial fibrillary acidic protein (GFAP) staining was also performed in healthy control and EAE mice. Increased astrocytosis was first observed at 9 dpi, subsequent to microglial activity, and also persisted until 33 dpi in both the retina and optic nerve of EAE mice (Fig. 4a and b). A strong appearance of astrogliosis was observed in the retina of EAE mice (Fig. 4b). The strong glial response in the retina was corroborated by the significantly higher mRNA expression of *Gfap* at 11 dpi (2-fold increase, *p* = 0.02), 15 dpi (7-fold increase, *p* < 0.00001), and 20 dpi (6-fold increase, *p* < 0.00001), while tapering off at 28 dpi (4-fold increase, *p* < 0.00001), and 33 dpi (3-fold increase, *p* = 0.0002) in EAE mice compared to healthy controls (Fig. 4c).

### Further inflammatory responses in EAE

**Fig. 5:**
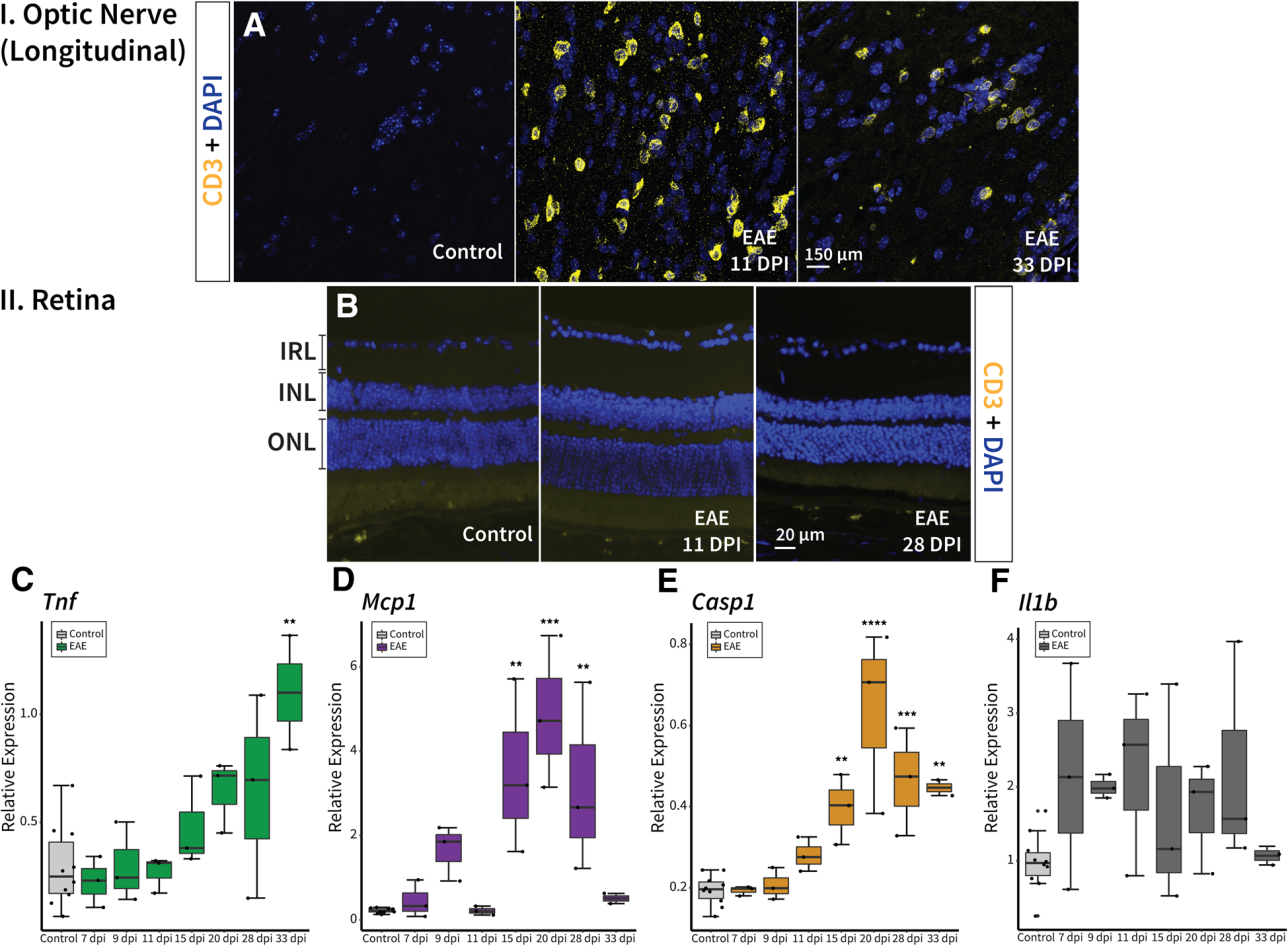
A strong inflammatory response was observed in the optic nerve and retina of EAE mice. **a** T-cells (CD3) initially appear 11 days post immunisation (dpi) along with the cellular infiltration (DAPI), subsiding at later time points but still remaining until 33 dpi in the optic nerve of EAE mice. **b** T-cells were not observed in the retina of EAE mice at any time point. Yet, **c** *Tnf* expression amplified from 20 to 33 dpi in EAE compared to healthy controls in the retina. *Tnf* is a proinflammatory cytokine involved in the innate immune response. Whereas, **d** mRNA expression of *Mcp1* in the retina increased significantly between 15 dpi and 28 dpi in EAE mice. *Mcp1* likely plays an amplifying role (rather than an initiating role) in EAE. **e** Caspase 1 (*Casp1*) increases significantly from 15 dpi to 33 dpi in EAE retinas. It has a crucial role in development of immune mediated inflammatory processes leading to central nervous system demyelination. However, **f** *Il-1β*, which is a cytokine activated by Caspase 1, was not different in retinal expression between EAE mice and healthy controls. *p*-values for comparison between EAE and control mice (**** *p* < 0.00001, *** *p* < 0.0001, ** *p* < 0.01). EAE: experimental autoimmune encephalomyelitis, TNF: tumor necrosis factor, MCP1: monocyte chemoattractant protein 1, IL-1β: interleukin-1β, IRL: inner retinal layer, INL: inner nuclear layer, ONL: outer nuclear layer. EAE: experimental autoimmune encephalomyelitis, IBA1: allograft inflammatory factor 1, TMEM119: transmembrane protein 119, IRL: inner retinal layer, INL: inner nuclear layer, ONL: outer nuclear layer

T-cell presence was investigated, since it plays a significant role in pathogenesis and tissue damage in animal models of MS [18]. First signs of T-cells (CD3) appeared at 11 dpi along with extensive cellular infiltration (observed via DAPI nuclei staining) in the optic nerves of EAE mice (Fig. 5a). Infiltrating cells appeared to accumulate near the ONH but were also observed throughout the optic nerve in EAE mice (Fig. 5a). T-cell presence diminished thereafter, but remained evident until 33 dpi in EAE mice (Fig. 5a). There was no sign of T-cells in the retina at any time point (Fig. 5b). Previous studies have also reported little to no T-cell or macrophage infiltration of the retina in these experimental ON models [45]. To explore other signs of inflammatory response in the retina, immune cell and inflammatory markers were analyzed with real-time PCR. We first examined expression of *Tnf* (tumor necrosis factor), which was amplified at 20 dpi (2-fold increase), 28 dpi (2-fold increase), and significantly increased at 33 dpi (4-fold increase, *p* = 0.001) in EAE compared to healthy controls (Fig. 5c). *Tnf* is activated by microglia as well as astrocytes and involved in acute-phase immune-mediated injury [46], which appears to play a role in EAE retinal pathology. Retinal expression of *Mcp1* (monocyte chemoattractant protein 1, also known as *Ccl2*; implicated in mediating monocyte recruitment) significantly increased at 15 dpi (10-fold increase, *p* = 0.001), and 20 dpi (14-fold increase, *p* = 0.00003), tapering off from 28 dpi (9-fold increase, *p* = 0.002) until 33 dpi in EAE mice (Fig. 5d). There was also a significant increase in *Casp1* (Caspase-1) expression at 15 dpi (2-fold increase, *p* = 0.004), 20 dpi (3-fold increase, *p* < 0.00001), 28 dpi (2.5-fold increase, *p* = 0.0004), and 33 dpi (2-fold increase, *p* = 0.003) in EAE retinas compared to healthy controls (Fig. 5e). Caspase-1 plays a pivotal role in the apoptotic cascade [47]. The mRNA expression of *Il1β* (Interleukin-1β), produced by microglia and is a target of caspase-1, was not statistically different at any time point (Fig. 5f).

### Demyelination and axonal pathology

**Fig. 6:**
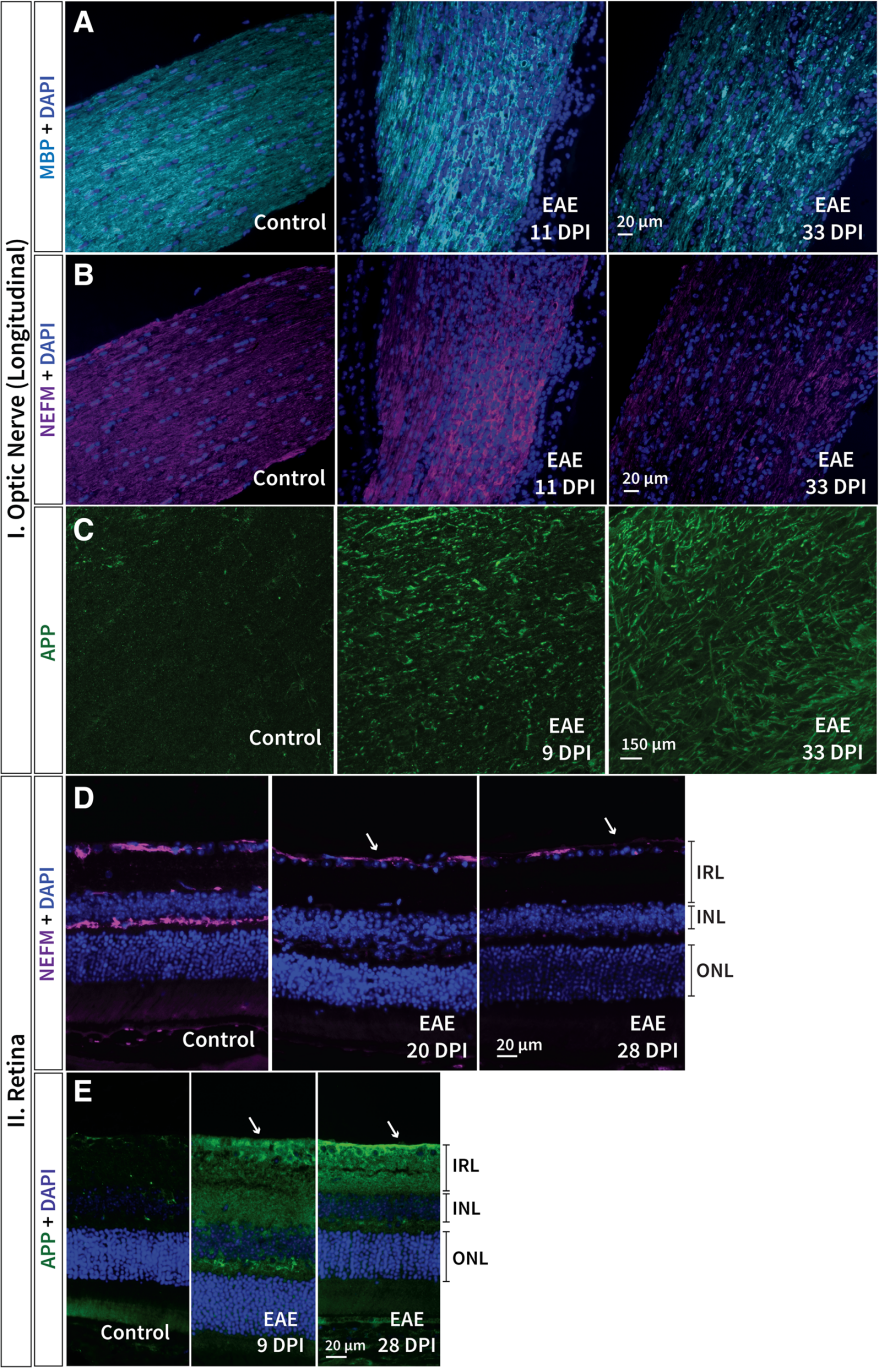
Demyelination and axonal pathology was perceived in both the retina and optic nerve in EAE. **a** Demyelination (MBP) in the optic nerve was first observed 11 days post immunisation (dpi) progressing until 33 dpi where profound signs of myelin disorganisation and possible myelin debris were observed. **b** Axonal degeneration (NEFM) was observed from 11 dpi until 33 dpi in the optic nerve of EAE mice. **c** APP, important for axonal transport, appears to increase as early as 9 dpi and also persisted until the final observational time point in EAE optic nerve sections. In the retina, **d** NEFM appears to decrease in the IRL (arrows) and in the outer plexiform layer over time in EAE mice; beginning later (20 dpi) than in the optic nerve. **e** APP in the retina appears to increase (arrows) as early as 9 dpi similar to presentation in the optic nerve for EAE mice. EAE: experimental autoimmune encephalomyelitis, MBP: myelin basic protein, NEFM: neurofilament-M, APP: Alzheimer precursor protein, IRL: inner retinal layer, INL: inner nuclear layer, ONL: outer nuclear layer

Initial signs of demyelination (loss of MBP), a key factor implicated in MS and EAE pathology, were observed in the optic nerve of EAE mice at 11 dpi progressing until 33 dpi, at which the greatest loss of immunoreactivity was detected (Fig. 6a). Furthermore, the observation of speckles at the final observational time point (dpi 33) in EAE mice might indicate myelin debris or disorganized remyelination following disruption (Fig. 6a). Neurofilament-M (NEFM) immunoreactivity decreased concurrently with MBP from 11 dpi until 33 dpi, associated with profound axonal degeneration in the optic nerve of EAE mice (Fig. 6b). In the retina, NEFM decrease in the IRL was observed later than in the optic nerve; beginning at 20 dpi and persisting until the final observational time point in EAE mice compared to healthy controls (Fig. 6d). NEFM decrease was also detected in the outer plexiform layer of EAE mice from 20 to 33 dpi (Fig. 6d). To assess other markers of axonal impairment, amyloid precursor protein (APP; involved in axonal transport) was investigated and observed to increase as early as 9 dpi and persevered until 33 dpi in both EAE optic nerves and retinas (Fig. 6c and e).

### Neurodegeneration and RGC deterioration

**Fig. 7:**
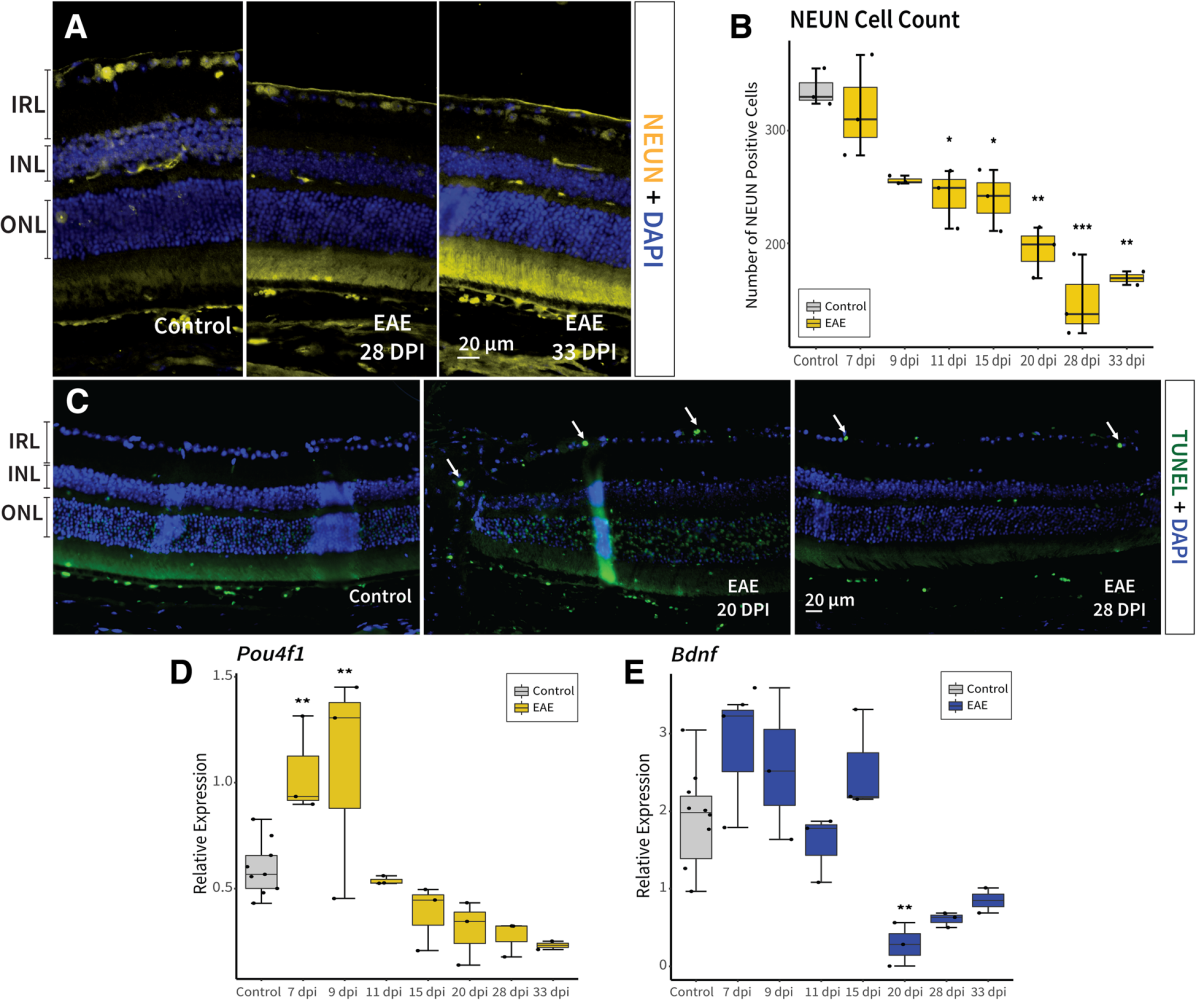
Neurodegeneration in the retina was observed throughout the observational period in EAE. **a**-**b** The number of NEUN positive cells decreased significantly from 11 days post immunisation (dpi) to 33 dpi in EAE mice compared to healthy controls. Furthermore, **c** apoptosis was observed using TUNEL staining from 20 to 33 dpi in the ganglion cell layer (arrows) of EAE mice. **d** *Pou4f1*, a transcription factor expressed in retinal ganglion cells first increased significantly at 7 and 9 dpi (possibly due to compensatory mechanisms within the cell) followed by a decrease from 20 to 33 dpi. **e** *Bdnf* retinal expression, which is an important neurotrophic factor for neuronal survival, also decreased at 20 and 28 dpi in EAE mice compared to healthy control, further exemplifying neurodegeneration. *p*-values for comparison between EAE and control mice (*** *p* < 0.0001, ** *p* < 0.01, * *p* < 0.05). EAE: experimental autoimmune encephalomyelitis, NEUN: neuronal nuclei, TUNEL: terminal deoxynucleotidyl transferase dUTP nick end labelling, IRL: inner retinal layer, POU4F1: POU domain class 4 transcription factor 1, BDNF: brain-derived neurotrophic factor, INL: inner nuclear layer, ONL: outer nuclear layer

Subsequent to demyelination and axonal degeneration, downstream RGC damage was expected [48]. Along these lines, there was a significant decrease in the number of NEUN positive cells labelling neurons (primarily RGCs) at 11 dpi (242.0 ± 26.2, *p* = 0.02), 15 dpi (239.3 ± 27.1, *p* = 0.02), 20 dpi (194.0 ± 22.9, *p* = 0.0004), 28 dpi (149.0 ± 36.5, *p* = 0.00002), and 33 dpi (169.0 ± 8.5, *p* = 0.0002) in EAE mice compared to healthy controls (336.3 ± 16.4; Fig. 7a/b). The presence of TUNEL positive cells in the ganglion cell layer (GCL; Fig. 7c) confirmed the occurrence of apoptosis at 15 dpi to 33 dpi in EAE mice. *Pou4f1* (POU class 4 homeobox 1, also known as *Brn3a*), a transcription factor expressed in neurons including RGCs [49], was first amplified significantly at 7 (2-fold increase, *p* = 0.01), and 9 dpi (2-fold increase, *p* = 0.01) – possibly due to compensatory mechanisms of the cell – then decreased from 20 (2-fold decrease) to 33 dpi (3-fold decrease) when damage had accumulated in EAE mice (Fig. 7d). *Bdnf* (brain-derived neurotrophic factor; important for neuronal survival [50]), decreased in expression significantly at 20 dpi (6-fold decrease, *p* = 0.03), and marginally at 28 (3-fold decrease) and 33 dpi (2-fold decrease) in EAE mice, further revealing the presence of a neurotoxic environment within the retina (Fig. 7e).

### Müller cell reactivity

**Fig. 8:**
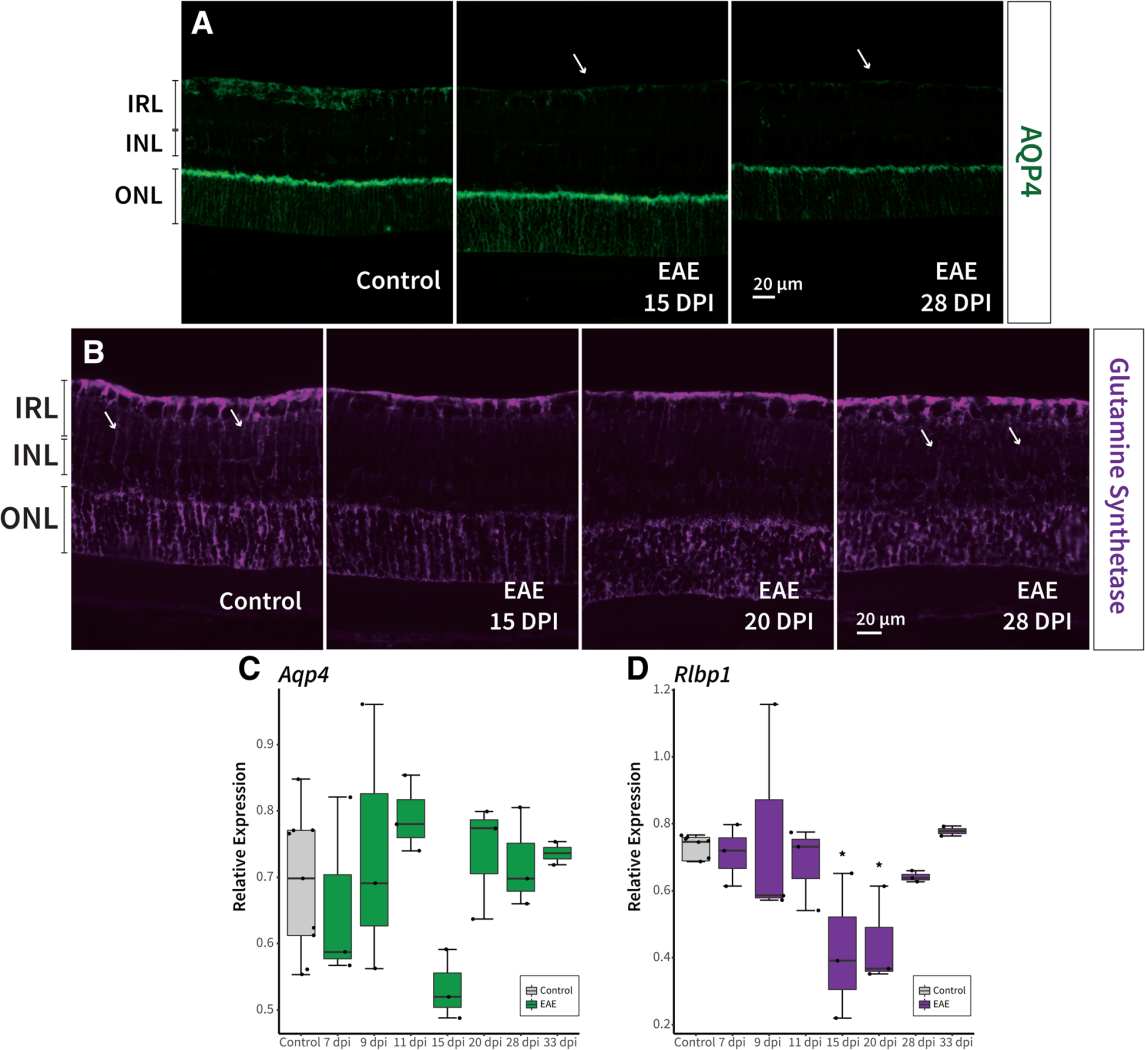
Müller cell reactivity was detected following signs of optic neuritis. **a** A decrease in AQP4 signal (arrows) in the retinal nerve fibre layer was observed 15 and 28 days post immunisation (dpi) in the retina of EAE mice. **b** Glutamine synthetase, expressed in Müller cells, decreased in the Müller cell branched processes at 15 and 20 dpi but appeared to recover by 28 dpi in EAE mice. Arrows in the healthy control retina point to intact Müller cell processes, while in EAE, the arrows indicate the recovered processes that appear disorganized. **c** mRNA expression of *Aqp4* decreased only at 15 dpi, while **d** *Rlbp1* expression decreased significantly at 15 and 20 dpi in EAE mice retinas compared to healthy controls. *p*-values for comparison between EAE and control mice (* *p* < 0.05). EAE: experimental autoimmune encephalomyelitis, AQP4: aquaporin-4, RLBP1: retinaldehyde-binding protein 1, IRL: inner retinal layer, INL: inner nuclear layer, ONL: outer nuclear layer

The role Müller cells play in EAE retinal pathology has been relatively unexplored. First, we assessed aquaporin-4 (AQP4; found abundantly on Müller cells) using immunostaining, which had a signal decrease at 15 and 28 dpi, specifically in the IRL of EAE mice compared to healthy controls (Fig. 8a). mRNA expression of *Aqp4* in the retina decreased specifically at 15 dpi (1.5-fold decrease) in EAE mice compared to healthy controls, however, this did not reach statistical significance after correction for multiple comparisons (*p* = 0.04 uncorrected, *p* = 0.3 corrected; Fig. 8c). The intensity of glutamine synthetase (GS) staining, also found on Müller cell processes, appeared to decrease at 15 and 20 dpi with some recovery by 28 dpi in EAE mice compared to healthy controls (Fig. 8b). Finally, *Rlbp1* (retinaldehyde binding protein 1, also known as *Cralbp*), expressed in Müller cells, significantly decreased in expression only at 15 (1.5-fold decrease, *p* = 0.05) and 20 dpi (1.5-fold decrease, *p* = 0.05) in EAE retinas (Fig. 8d).

### Histological assessment

**Fig. 9:**
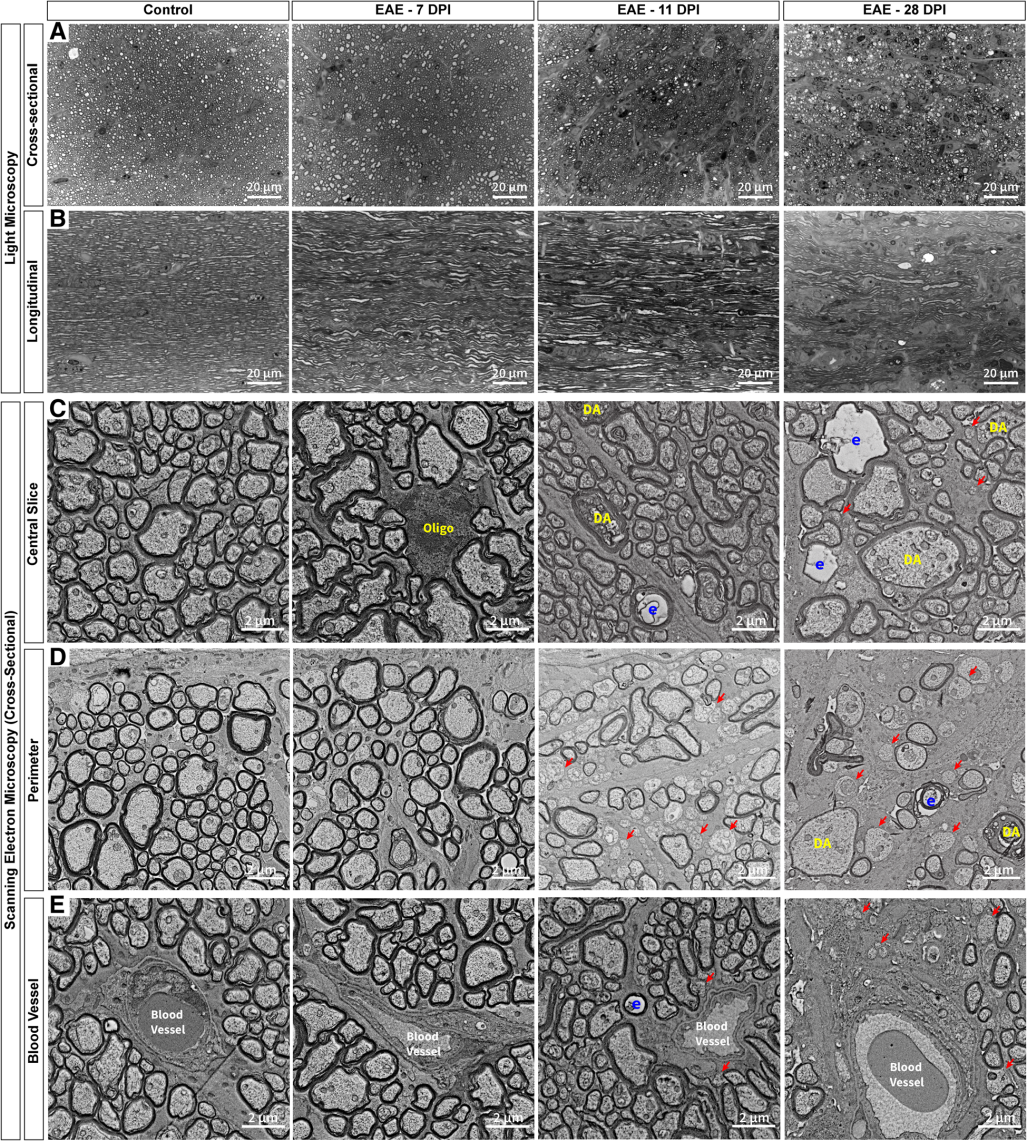
Optic nerve pathology began near the perimeter and blood vessels and progressed into the center. In the optic nerve **a**-**b**, the fibres that surround the axon bundles appear to be disrupted by 11 days post immunisation (dpi) in EAE mice. **a** There was also a reduction in the number of axons present in EAE mice compared to healthy controls at 11 dpi and 28 dpi in the optic nerve. **b** In the longitudinal optic nerve sections, an overall disruption of axonal organisation was observed in EAE mice. **c**-**e** Scanning electron micrographs of cross-sectional optic nerves in healthy and EAE mice provide a closer look. **c** In central sections, signs of axonal degeneration were first observed 11 dpi, while at 28 dpi severe pathology was present throughout the entire optic nerve. **d** Near the perimeter of the optic nerve sections, strong axonal degeneration was observed at both 11 and 28 dpi in EAE mice compared to healthy controls. Severe loss of axons and large empty spaces were also observed at 28 dpi near the perimeter of the optic nerve. **e** Some demyelinated axons were observed along with axonal degeneration at 11 dpi near blood vessels, while at 28 dpi severe axonal loss was present in EAE mice compared to healthy controls. Red arrows: demyelinated axons, DA: degenerating axon, E: empty myelin sheath with no axon, Oligo: oligodendrocyte, EAE: experimental autoimmune encephalomyelitis. Electron micrograph image resolution: 8 nm/pixel

Since signs of axonal degeneration (i.e. NEFM and APP staining) and demyelination (i.e. MBP staining) was observed in the optic nerve, histological morphology was evaluated with light and electron microscopy at 7, 11, and 28 dpi. In cross-sectional optic nerve sections, the pial septa that surrounds the axon bundles appears to be disrupted at both 11 and 28 dpi in EAE mice (Fig. 9a). Furthermore, irregular myelination, thinner myelin sheaths, and a high proportion of unmyelinated axons can be observed at 28 dpi in EAE mice (Fig. 9a) similar to the demyelination observed with MBP staining (Fig. 6b). In the longitudinal optic nerve sections, a disruption of axonal organization was observed, and the space within the axons appeared to be enlarged at 11 and 28 dpi (Fig. 9b). The scanning electron micrographs of the optic nerve revealed more detailed insight into the pathological changes observed in EAE mice. In healthy controls, nerve fibres were densely packed with a combination of large and small fibres throughout the optic nerve (Fig. 9c-e). At 11 dpi, optic nerve pathology (including demyelination and degenerated axons) appeared to begin near the perimeter of the optic nerve cross-sections (Fig. 9d) and to a lesser extent around the blood vessels (Fig. 9e). By 28 dpi, severe degeneration was observed completely from the perimeter to the centre of the optic nerve in EAE mice (Fig. 9c). The early pathology at the perimeter of the optic nerve cross-sections may be caused by inflammatory cells such as T-cells passing from the cerebral spinal fluid (which surrounds the entirety of the optic nerve) through the pial septa, which has also been observed in ON patients [51]. Infiltrating immune cells can also contribute to local pathology around the blood vessels following vascular leakage.

**Fig. 10:**
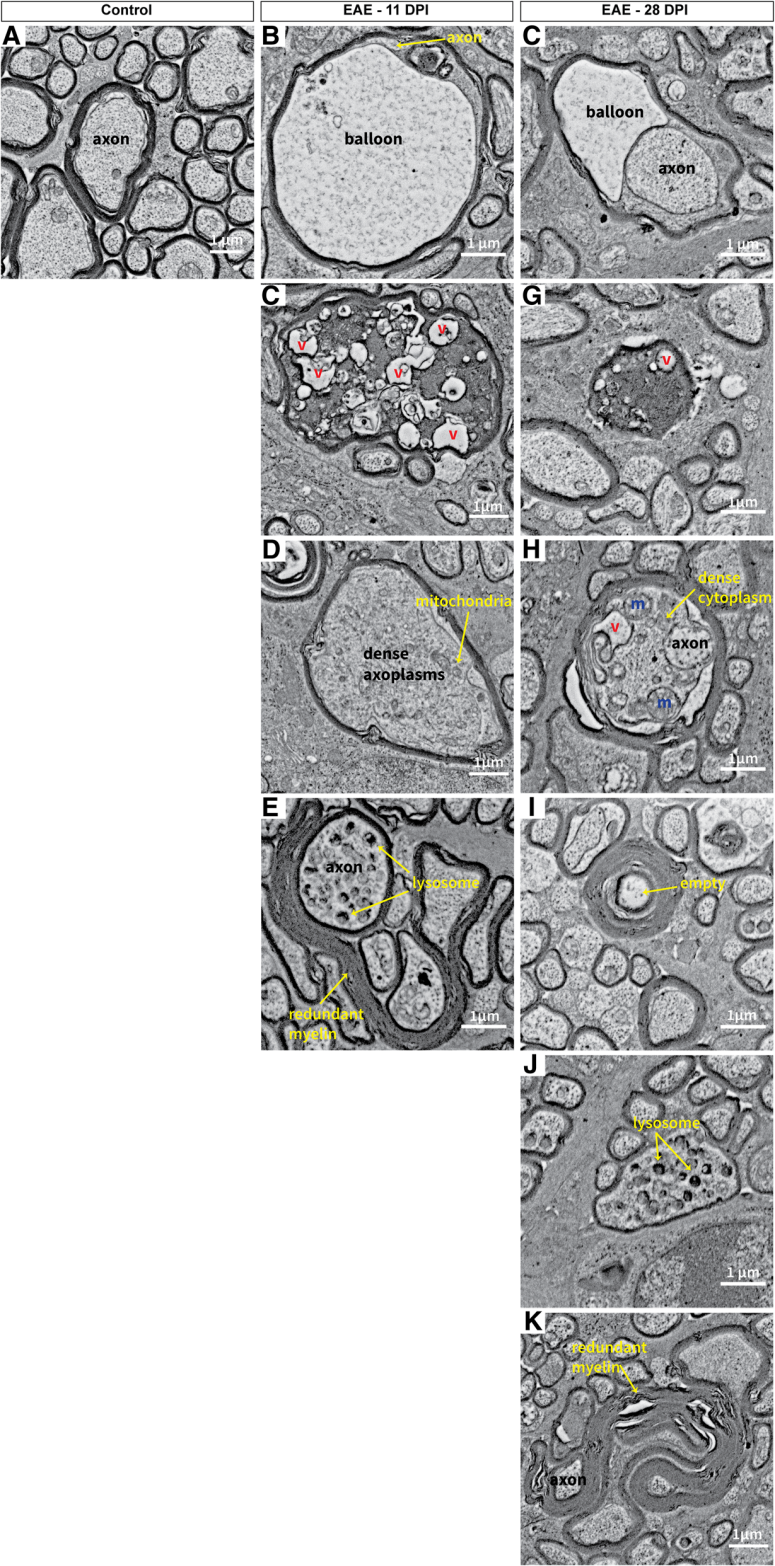
Examples of optic nerve pathology observed in EAE mice 11 and 28 days post immunisation. **a** Example of a healthy axonal fibre with normal myelination. **b** Swollen nerve fibres with a fragment of axon left and a large balloon produced from the splitting of the myelin sheaths was observed in EAE mice at 11 dpi. **c** Another large swollen nerve fibre with dark cytoplasm (likely a sign of severe degeneration) filled with numerous vacuoles at 11 dpi. **d** Many bloated myelinated axons were observed at 11 dpi with a dense axoplasm filled with primarily neurofilament. **e** A dystrophic axon where the axoplasm contains an accumulation of lysosomes. Redundant myelination can also be found around the axons at 11 dpi. **f** In EAE mice at 28 dpi, ballooning was also observed in some nerve fibres. **g** Nerve fibres with dark cytoplasm were identified at 28 dpi as well but appeared more shrunken than at 11 dpi. **h** At 28 dpi, large myelinated nerve fibres showed dense degeneration with numerous vacuoles and organelles within the cytoplasm, however they were not as large as fibres at 11 dpi. **i** Empty myelin sheaths with completely degenerated axons were visible at 28 dpi in EAE mice. **j** Dystrophic axons were also found at 28 dpi. **k** Redundant myelin was more common at 28 dpi, often forming around tiny fragments of axons or entirely on its own. v: vacuoles, m: mitochondria, EAE: experimental autoimmune encephalomyelitis. Electron micrograph image resolution: 8 nm/pixel

While, there was heterogeneity in the EAE mice at 11 dpi, a substantial number of optic nerve fibres exhibited signs of swelling (Fig. 10b-d) compared to healthy controls (Fig. 10a). Numerous fibres with large myelin balloons, likely filled with fluid, were found at 11 dpi (Fig. 10b). Similarly at 28 dpi, myelin ballooning was observed, although the effect was less pronounced than at 11 dpi (Fig. 10f). Initial signs of axonal degeneration included accumulation of mitochondria and lysosomes within the axoplasm of several axons (Fig. 10e/h/j), with considerably more nerve fibres affected by 28 dpi. In some cases, accumulation of neurofilaments could be observed in the dense axoplasm of fibres, indicating another form of axonal degeneration (Fig. 10d). Nerve fibres with dark cytoplasm indicated even further deterioration of the axons, which appeared enlarged with vacuoles at 11 dpi (Fig. 10c) and shrunken at 28 dpi (Fig. 10g). Numerous fibres with empty myelin sheaths were found at 28 dpi (Fig. 10i) indicating complete axonal degeneration. Usually these axons were surrounded by thicker than normal myelin sheaths, likely a product of repeated remyelination in an attempt to repair (Fig. 10i). This remyelination was redundant and defective in the EAE optic nerve at 11 and 28 dpi (Fig. 10e/k) and may possibly be associated with the myelin speckles observed in Fig. 6a.

No gross notable changes were observed in the retinal morphology using light microscopy in EAE mice (Additional file 2A) contrasting the OCT-derived IRL thickness changes observed in EAE mice. A possible explanation is that fixation by default decreases fluids, therefore, it is difficult to observe and evaluate swelling using histological assessment. Previous studies have also observed little evidence for alterations in histological retinal thickness in EAE mice including measurements of the total retinal thickness, inner plexiform layer, INL and OPL [9, 52]. Nonetheless, electron microscopy of the retina showed neuronal cell bodies with dark cytoplasm and numerous vacuoles (likely indicating degeneration) in the GCL at 11 and 28 dpi in EAE mice (Additional file 2B), corroborating the findings of apoptosis with TUNEL staining (Fig. 7c).

## Discussion

The aim of this study was to add morphological substrate to OCT-derived retinal measurements using ex-vivo analysis of retinal and optic nerve tissue and to explore the biological mechanisms of visual pathway damage in EAE mice. Fitting with previous assumptions, inflammation and edema were largely associated with IRL thickening at clinical onset of experimental ON. Signs of early retinal pathology (i.e. APP increase and changes in *Pou4f1* expression) were observed, and preceded IRL thinning at the earlier time points. In fact, the inflammatory edema-induced IRL thickening may overshadow any thinning generated by early retinal degeneration, at least until inflammation subsides partially and the accumulation of sustained neuro-axonal damage becomes considerable. The early glial response (including microglia and astrocyte activation) in the retina prior to inflammatory demyelination in the optic nerve may contribute to primary retinal pathology.

Furthermore, Müller cell reactivity was observed in the IRL, subsequent to early astrogliosis and recovered at later time points. It is also clear that rapid retrograde degeneration following ON played a substantial role in IRL thinning: damage in the optic nerve was followed by signs of axonal degeneration (i.e. NEFM decrease) in the retina. Finally, the accumulating effects of the sustained neuro-axonal damage, inflammatory mediated demyelination, and glial pathology likely contributed to the significant IRL thinning observed at the final observational time point.

### Inflammatory edema substantially contributes to IRL thickening at clinical onset of experimental ON

The increased IRL thickness at early time points (Fig. 2a) coincides with the significant inflammatory response observed in both the retina and optic nerve. Early microglial activation and astrogliosis in both the retina and optic nerve (Figs. 3-4), followed by extensive cellular infiltration (including T-cells) in the optic nerve at 11 dpi (Fig. 5), likely further amplified the inflammatory process within the retina, potentially contributing to the acute IRL thickening. Since astrogliosis was present in EAE mice, it is possible that astroglial end-feet dysfunction can result in retinal water imbalance and BRB impairment (observed concurrently at 9 dpi; Additional file 1), which has also been associated with vasogenic edema [53, 54]. Furthermore, impaired astrocyte end-feet and the swollen cell bodies can result in extensive gaps in the pial septa and glial limitans, as observed in EAE optic nerve sections at 11 dpi (Fig. 9). Disruption to the connective tissue and glial fibers that surround the axon bundles may also add to optic nerve swelling via extracellular edema. The inflammatory changes in the orbital subarachnoid spaces of the ONH could contribute to the edema. Since the ONH lacks the classical characteristics of the blood-brain-barrier, it can allow non-specific permeability and be more vulnerable to damage [51]. Infiltration of peripheral inflammatory cells at 11 dpi in the optic nerve were predominantly observed near the ONH, possibly contributing to swelling of the IRL observed on OCT. Also, it is feasible that optic nerve axonal swelling (as observed by electron micrographs at 11 dpi) could propagate backwards into the retina contributing to the increased IRL thickness observed at 11 dpi in EAE mice. A combination of both edema and a strong inflammatory response likely resulted in the overall IRL thickening observed early in EAE.

### Early retinal pathology prior to cellular infiltration or immune-mediated demyelination of the optic nerve

While demyelination in the optic nerve occurred concurrently with axon damage at 11 dpi, it does not seem to be a prerequisite for retinal neuro-axonal degeneration in EAE mice. The increase in APP signal indicated early axonal transport impairment in both the retina and optic nerve, preceding the loss of structural integrity to the axons themselves as reflected by the decrease in NEFM (Fig. 6). Moreover, *Pou4f1* upregulation in the early stages of EAE (possibly occurring below a certain threshold of neuronal damage) might activate endogenous compensatory mechanisms within RGCs and is another sign of early RGC pathology (Fig. 7d). Previous studies have also reported RGC loss prior to inflammatory infiltration, demyelination, or edema of the optic nerve [17, 55–57]. In line with reports by other authors [3], we found that early signs of RGC pathology precedes IRL thinning, suggesting that neuronal damage may occur, at least partially, independent of apparent inflammation. Despite the clear indication of early neuro-degenerative processes occurring in EAE, the net initial structural response was IRL thickening. This apparent discrepancy is probably a consequence of the severe inflammatory response and subsequent swelling, outweighing the contribution of early stage neurodegeneration. Once inflammation and edema have subsided, the effects of ongoing neuro-axonal degeneration may manifest as a reduction of IRL thickness.

### Retrograde degeneration plays a significant role in IRL thinning

The observed temporal dynamics of RGC damage suggest that secondary neuro-axonal injury (following inflammation-induced demyelination) is a principal mechanism involved in retinal pathology. In particular, axonal damage (assessed by NEFM) appeared later in the retina compared to the optic nerve (Fig. 6). Likewise, DNA degradation (TUNEL staining), indicative of cell loss via apoptosis, was observed only at later time points (Fig. 7c). Electron microscopy cross-sectional images provided further support for retrograde degeneration; indicating significant deterioration in the optic nerve, beginning at the perimeter (11 dpi) and progressing into the centre by 28 dpi, where the highest density of axonal bundles exists (Fig. 9). In line with our findings, previous studies suggested that axonal damage, possibly via retrograde degeneration, results in apoptosis of RGCs after clinical manifestation of the disease [2, 45, 51, 56]. Thus, retrograde degeneration following ON is likely an important factor contributing to IRL thinning observed at the final time points.

### Neuro-axonal degeneration throughout the entire disease course contributes to IRL thinning at later stages

In addition to retrograde degeneration, overall IRL thinning in EAE mice is seemingly further enhanced by a net combination of the sustained neuro-axonal degeneration, inflammatory-mediated demyelination, and glial pathology. Once significant sections of myelin or axon have deteriorated, the RGC body itself is likely to die, and the decrease in OCT-derived IRL thickness at later time points might reflect neuronal in addition to axonal degeneration. Although signs of remyelinated optic nerve axons were observed (Fig. 6a/10), it appeared irregular and redundant in nature. The observed myelin debris can be processed through the autophagy-lysosome pathway, promoting further inflammation, [58] possibly contributing further to the fibrotic scar formation that was observed in the optic nerve (Fig. 9). Additional IRL damage was likely promoted by increased expression of *Casp1* (Fig. 5e) and decreased expression of *Bdnf* (Fig. 7e) in EAE at later time points. Since BDNF is involved in anterograde fast axonal transport [59], the reduction in *Bdnf* expression observed in our study may contribute to additional axonal transport failure in EAE mice. Damage in the early phase of the disease (likely driven by a local inflammatory response, i.e. astrocytes and resident microglia) compounds with late stage disease activity (i.e. accumulating inflammatory effect and further primary and secondary neuro-axonal damage), thereby accelerating IRL thinning in the late stages of the disease.

### Gliosis observed prior to immune-mediated demyelination and its involvement in early retinal pathology

There was evidence for an early inflammatory response from resident microglial cells (stained with TMEM119) observed prior to vascular leakage in EAE (Fig. 3). A significantly greater number of microglial cells were present in the optic nerve than in the retina, likely due to the optic nerve being one of the primary sites of injury in this experimental ON model. Previous studies suggested that resident microglia was only weakly activated, whereas infiltrating macrophages are highly immunoreactive [60]. This is complementary to the current findings of a weaker microglial response in the retina compared to the optic nerve, as there was a lack of evidence for cellular infiltration (including T-cells) in the retina at any time point (Fig. 5b).

Since early signs of astrogliosis were also detected subsequent to microglial activation, it is possible that astrocytes were activated by resident microglial cells prior to signs of ON and cellular infiltration. Previous studies have also suggested that CD68+ activated microglia and macrophages (also observed in this study, not shown) could be potent inducers of the A1 astroglial phenotype; important for the pro-inflammatory response [53]. Another possibility is astrocyte activation via damage to the end-foot processes that surround the vasculature, or as a result of signaling products from the periphery released into the retina following albumin leakage, detected concurrently at 9 dpi in the retina (Additional file 1). Earlier studies have also observed BRB leakage early in the disease course [2, 61–63]. It is likely a combination of both microglial activation and vascular abnormalities that initiate the early astrocytic response in the retina.

This initial local inflammatory response could contribute to the observed primary retinal pathology. Activated microglia quickly proliferate and engage in phagocytosis of cellular debris, which may be a response to early retinal disturbances but can also generate further inadvertent primary pathology [64]. It has been suggested that the production of oxidative species by astrocytes, along with other inflammatory mediators, may contribute to neuro-axonal damage [65]. *Tnf* expression which increased in EAE mice (Fig. 5c), can contribute to synaptic deficits in addition to inducing neuronal cell death by controlling the release of glutamate from astrocytes [66–69]. While, *Mcp1* (Fig. 5d), that may be released by both astrocytes and microglia, is involved in mediating immunopathology and likely has an amplifying, rather than an initiating role [67, 70–72], promoting further retinal damage.

### Müller cell reactivity during EAE retinal pathology

Müller cells appeared to undergo location- and time-specific structural changes in EAE mice, as reflected by a decrease in *Rlbp1* mRNA expression and GS immunostaining in EAE mice at 15 and 20 dpi. Since the basal end-feet of Müller cells form adherent junctions with astrocytes, the previously noted early astroglial pathology in the retina may also induce Müller cell reactivity at these later time points. Inflammatory mediated demyelination of the optic nerve was observed at 11 dpi prior to Müller glial susceptibility, thus it is also possible that Müller cells are responding to this inflammation and early RGC pathology. Aquaporin-4 is a water channel located on Müller cells near the BRB [18, 73], and decreased expression in EAE mice at 15 dpi along with *Rlbp1*, further supports the time specific changes observed in Müller cells. Müller cells may react to the swelling observed at 11 dpi (Fig. 2a/10) by a subsequent downregulation of *Aqp4* as a protective mechanism against uncontrolled propagation of edema [74]. While GS, found primarily on Müller cells, is involved in regulating extracellular glutamate levels, and disruption of this process can result in excitotoxicity of ganglion cells [75]. Müller cells appear to respond to optic nerve injury by rapidly translocating GS to the region closest to the site of injury [75]. The decrease in GS signal also likely indicates the inability of this retinal glia to sustain its physiological maintenance role. Once the inflammatory response subsided at later time points (Figs. 3-5), Müller cell reactivity also decreased as observed by the recovery in *Rlbp1* expression and GS immunostaining (Fig. 8). In terms of locality, GS immunostaining revealed that these changes were most predominantly located in the Müller cell processes that span the inner plexiform layer (Fig. 8b), while, aquaporin-4 loss was observed specifically in the pRNFL (Fig. 8a). Since retinal pathology in this experimental ON model was primarily observed in the IRL, Müller cells may be more reactive in this region. Nonetheless, Müller glia disruption could contribute to the overall decrease in IRL thickness observed over time, since they are involved in modulating neuronal activity, maintaining retinal homeostasis, and providing metabolic support to RGCs [75].

INL, similar to previous reports [29], and OPL thickness did not change over time in EAE mice compared to healthy controls. In MS patients, INL thickening has been observed and is usually associated with ON or microcystic macular edema [76, 77]. This thickening is speculated to be due to intracellular edema caused by Müller mediated AQP4 reactivity or KIR4.1 dysfunction [78, 79], however, direct evidence for this has not been confirmed in the mouse model of MS. The lack of OCT-derived INL or OPL thickness difference in EAE may be because these layers are especially thin and difficult to accurately segment in mice (since current methods of segmentation involve a significant amount of manual correction). It may also indicate that pathology in this experimental ON model is primarily restricted to the IRL, including the IRL-specific Müller cell reactivity observed here. Taken together, these data provide new evidence for Müller cell reactivity that is 1) temporally specific to 15 and 20 dpi with signs of recovery at later time points, and 2) spatially specific to the IRL; responding to early inflammation and subsequent RGC pathology with compensatory mechanisms.

### Limitations

A small sample size of animals was assessed at each time point for the ex-vivo analysis. Therefore future studies should evaluate the significant findings from this study with more replicates to further confirm the results observed. Our study lacked a CFA-only control group to assess whether the effects of neuro-axonal degeneration observed are MOG-specific. However, previous studies assessing optic nerve and retinal pathology in autoimmune ON reported little to no difference between the CFA-only group and healthy controls in inflammatory pathology and neuro-axonal degeneration [2, 57]. Our results suggests that IRL changes in EAE are primarily influenced by pRNFL changes over time; yet previous OCT studies investigating pRNFL changes in EAE mice report contradictory findings [3, 32]. This may be due to the use of different species and immunisation protocols, but also because of methodological limitations as the pRNFL is a relatively thin structure in rodents and thus difficult to segment accurately on its own. More recent studies, including this report, addressed this problem by aggregating the pRNFL, GCL and inner plexiform layer into one structure, the IRL [11, 16, 31]. This may provide a more robust measure of EAE-induced pathology and has been reported to have excellent test-retest reliability [11].

## Conclusion

This descriptive study provides cellular and molecular insight into the possible mechanisms leading to ON and underlying structural changes of the retina in EAE mice. Findings in this mouse model correspond nicely with structural changes observed in patients suffering from MS-related acute-ON [6]. The initial transient IRL thickening coincides with signs of edema and with the significant inflammatory response detected at clinical onset of experimental ON. Effects of early retinal damage (prior to inflammation mediated demyelination in the optic nerve) on OCT-derived IRL measurements were likely outweighed by the significant swelling at earlier time points. Yet, the occurrence of early retinal degenerative pathology highlights the need for neuroprotective or -regenerative treatment of ON, since interventions applied at a later, or more chronic stage may not revert the possibly irreversible neuro-axonal damage that occurred initially. Pre-clinical trials targeting inflammatory mechanisms of retinal damage may consider 11 dpi as a useful OCT study check-point. Although retrograde mediated damage following ON appears to be a major mechanism leading to RGC degeneration, early pathology due to microglial activation, and astrocytosis may have contributed to IRL atrophy as well. Early inflammation appears to instigate Müller cell reactivity that may provide support to the retina during injury. Future studies should explore the neuroprotective role of Müller cells in experimental ON. Additionally, mechanistic studies assessing the role of Müller cells should be performed in the context of both MS, but also neuromyelitis optica spectrum disorder, where AQP4-mediated Müller pathology has been implicated [80, 81]. Following a reduction of the inflammatory response, neuro-axonal impairment became more visible in OCT readouts: earlier retinal damage accumulated with continued neuro-axonal degeneration and resulted in the severe IRL thinning observed at the final observational time point. These late OCT time points are suitable for testing interventions specifically targeting neurodegeneration. Moreover, the results in this study may be useful for sample size calculations with these end points in mind. Prospective studies may assess the relatively unexplored outer retinal layers in EAE, which have been reported to be dysfunctional in MS patients even in the absence of structural damage [82]. Overall, OCT measures of changes in IRL thickness in the mouse model of experimental ON can be rationalized in the context of cellular and molecular changes related to neuro-axonal pathology derived from immunohistochemical analysis. Furthermore, OCT may be useful for assessing pharmacological responses in pre-clinical trials for treatments targeting neurodegeneration in MS.

## Additional files

Additional file 1: *Vascular leakage was observed early in the retina of EAE mice.* Signs of vascular leakage in the retina were observed at both 9 and 11 dpi in EAE mice compared to healthy controls but were not seen at earlier or later time points. (PDF 6155 kb)

Additional file 2: *Retinal degeneration was observed in the ganglion cell layer in EAE mice.* (A) No notable gross changes were observed in EAE retinal light micrographs (B) scanning electron micrographs of retinal sections in healthy and EAE mice. In the retina, degeneration of cell bodies in the ganglion cell layer was observed at 11 and 28 dpi. (PDF 5034 kb)

## Data Availability

The datasets used and/or analysed during the current study are available from the corresponding author on reasonable request.

## Abbreviations

APP: Amyloid precursor protein
AQP4: Aquaporin 4
BDNF: Brain-derived neurotrophic factor
BRB: Blood retinal barrier
CASP1: Caspase 1
CFA: Complete Freund’s adjuvant
CNS: Central nervous system
DAPI: 4′, 6-diamidino-2-phenylindole
dpi: days post immunisation
EAE: Experimental autoimmune encephalomyelitis
GCL: Ganglion cell layer
GFAP: Glial fibrillary acidic protein
GS: Glutamine synthetase
IBA1: Allograft inflammatory factor 1
IL1β: Interleukin-1β
INL: Inner nuclear layer
IRL: Inner retinal layer
MBP: Myelin basic protein
MCP1: Monocyte chemoattractant protein 1
MOG: Myelin oligodendrocyte glycoprotein
MS: Multiple sclerosis
NEFM: Neurofilament-M
NEUN: Neuronal nuclei
OCT: Optical coherence tomography
ON: Optic neuritis
ONH: Optic nerve head
OPL: Outer plexiform layer
PBS: Phosphate-buffered saline
PCR: Polymerase chain reaction
POU4F1: POU class 4 homeobox 1
pRNFL: Peripapillary retinal nerve fiber layer
RGC: Retinal ganglion cell
RLBP1: Retinaldehyde blinding protein 1
TMEM119: Transmembrane protein 119
TNF: Tumor necrosis factor
TUNEL: Terminal deoxynucleotidyl transferase mediated dUTP nick-end labelling
