# Correction to: Retinal pathology in experimental optic neuritis is characterized by retrograde degeneration and gliosis

**DOI:** 10.1186/s40478-019-0825-0

**Published:** 2019-10-18

**Authors:** Praveena Manogaran, Marijana Samardzija, Anaïs Nura Schad, Carla Andrea Wicki, Christine Walker-Egger, Markus Rudin, Christian Grimm, Sven Schippling

**Affiliations:** 1 Department of Information Technology and Electrical Engineering, Swiss Federal Institute of Technology, Zurich, Switzerland; 2 Neuroimmunology and Multiple Sclerosis Research, Clinic for Neurology, University Hospital Zurich and University of Zurich, Zurich, Switzerland; 3 Department of Ophthalmology, Lab for Retinal Cell Biology, University of Zurich, Zurich, Switzerland; 4 Department of Biology, University of Zurich, Zurich, Switzerland; 5 Department of Health Sciences and Technology, Swiss Federal Institute of Technology, Zurich, Switzerland; 6 Institue for Biomedical Engineering, Swiss Federal Institute of Technology and University of Zurich, Zurich, Switzerland; 7 Institute of Pharmacology and Toxicology, University of Zurich, Zurich, Switzerland

**Correction to: Acta Neuropathol Commun (2019) 7:116**

**https://doi.org/10.1186/s40478-019-0768-5**

In the original publication of this article [1], figure 10 contained two panels “C” as panel “F” was accidentally omitted. The incorrect (Fig. 1) and correct (Fig. 2) versions are published in this correction article.

**Fig. 1:**
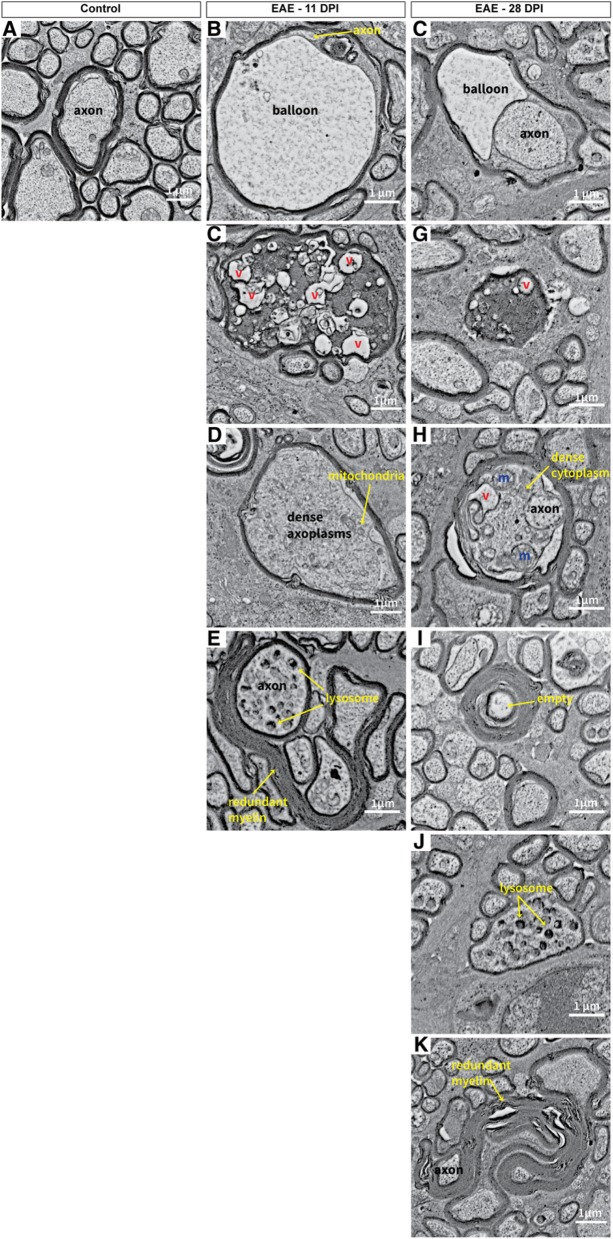
original version of figure 10

**Fig. 2:**
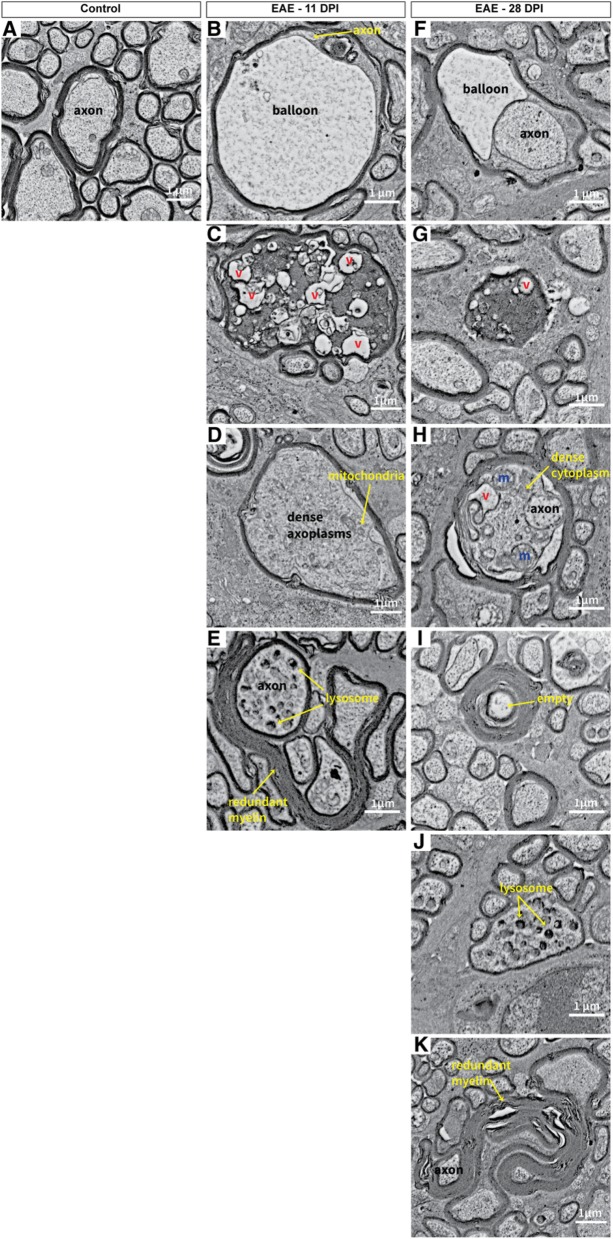
Correct version of figure 10
