# Supplementary material for: Retinal pathology in experimental optic neuritis is characterized by retrograde degeneration and gliosis
Source: Acta Neuropathol Commun. 2019 Jul 17;7:116. doi: 10.1186/s40478-019-0768-5 (PMC6637505; doi:10.1186/s40478-019-0768-5)
Supplement: Supplementary file 1 — Vascular leakage was observed early in the retina of EAE mice. Signs of vascular leakage in the retina were observed at both 9 and 11 dpi in EAE mice compared to healthy controls but were not seen at earlier or later time points. (PDF 6155 kb) [file 40478_2019_768_MOESM1_ESM.pdf]

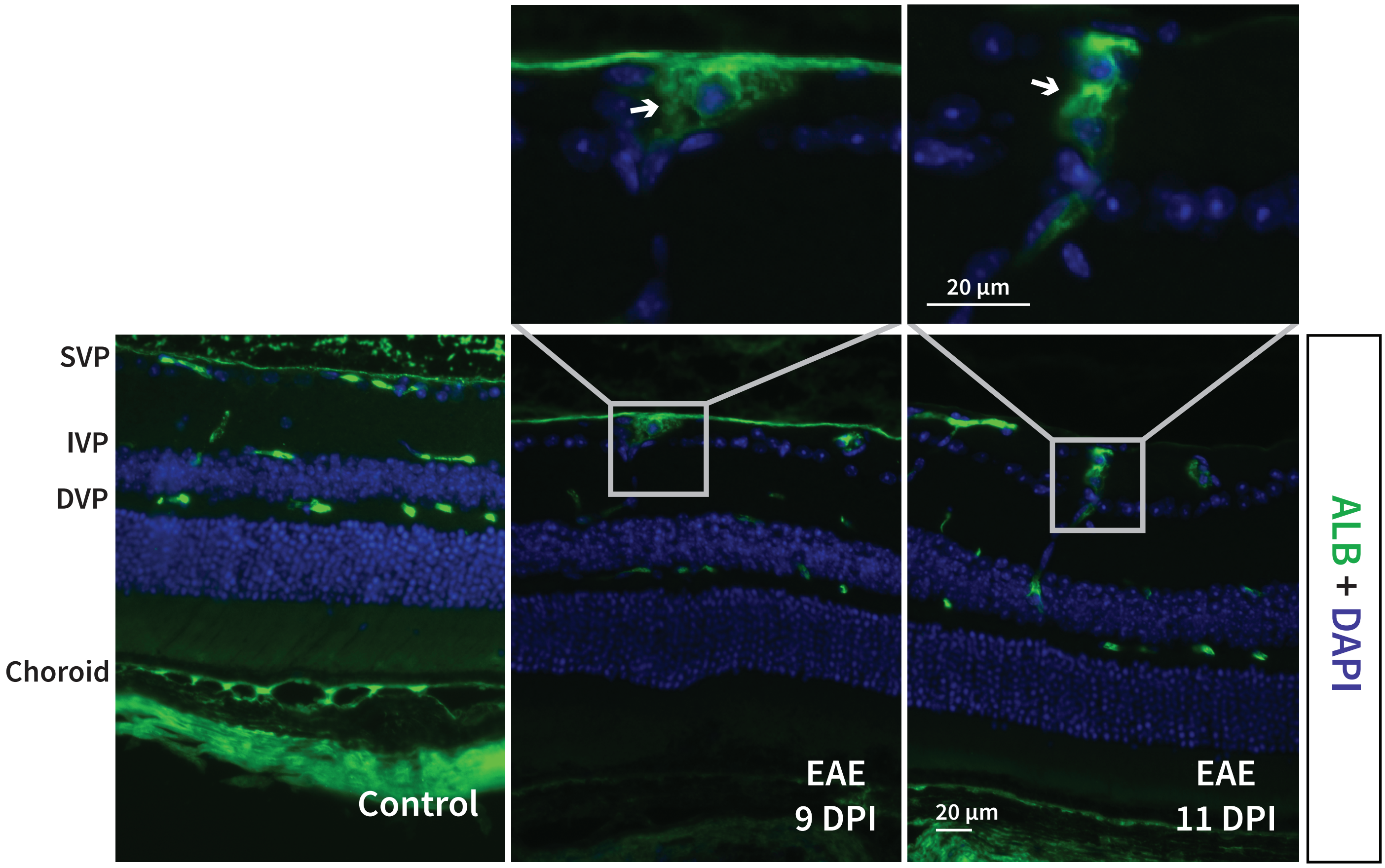

Additional File 1. Vascular leakage was observed early in the retina of EAE mice.
Signs of vascular leakage (ALB; arrows) in the retina were observed at both 9 and 11 dpi in EAE mice compared to healthy controls but were not seen at earlier or later time points.

DPI: days post immunisation, SVP: superficial vascular plexus, IVP: intermediate vascular plexus, DVP: deep vascular plexus, EAE: experimental autoimmune encephalomyelitis, ALB: albumin.

Retinal pathology in experimental optic neuritis is characterized by retrograde degeneration and gliosis.
Praveena Manogaran*[1,2], Marijana Samardzija, Anaïs Nura Schad, Carla Andrea Wicki, Christine Walker-Egger, Markus Rudin, Christian Grimm, Sven Schippling.
[1]Department of Information Technology and Electrical Engineering, Swiss Federal Institute of Technology, Zurich, Switzerland
[2]Neuroimmunology and Multiple Sclerosis Research, Clinic for Neurology, University Hospital Zurich and University of Zurich, Zurich, Switzerland
*pmanogar@student.ethz.ch
