# Supplementary material for: Retinal pathology in experimental optic neuritis is characterized by retrograde degeneration and gliosis
Source: Acta Neuropathol Commun. 2019 Jul 17;7:116. doi: 10.1186/s40478-019-0768-5 (PMC6637505; doi:10.1186/s40478-019-0768-5)
Supplement: Supplementary file 2 — Retinal degeneration was observed in the ganglion cell layer in EAE mice. (A) No notable gross changes were observed in EAE retinal light micrographs (B) scanning electron micrographs of retinal sections in healthy and EAE mice. In the retina, degeneration of cell bodies in the ganglion cell layer was observed at 11 and 28 dpi. (PDF 5034 kb) [file 40478_2019_768_MOESM2_ESM.pdf]

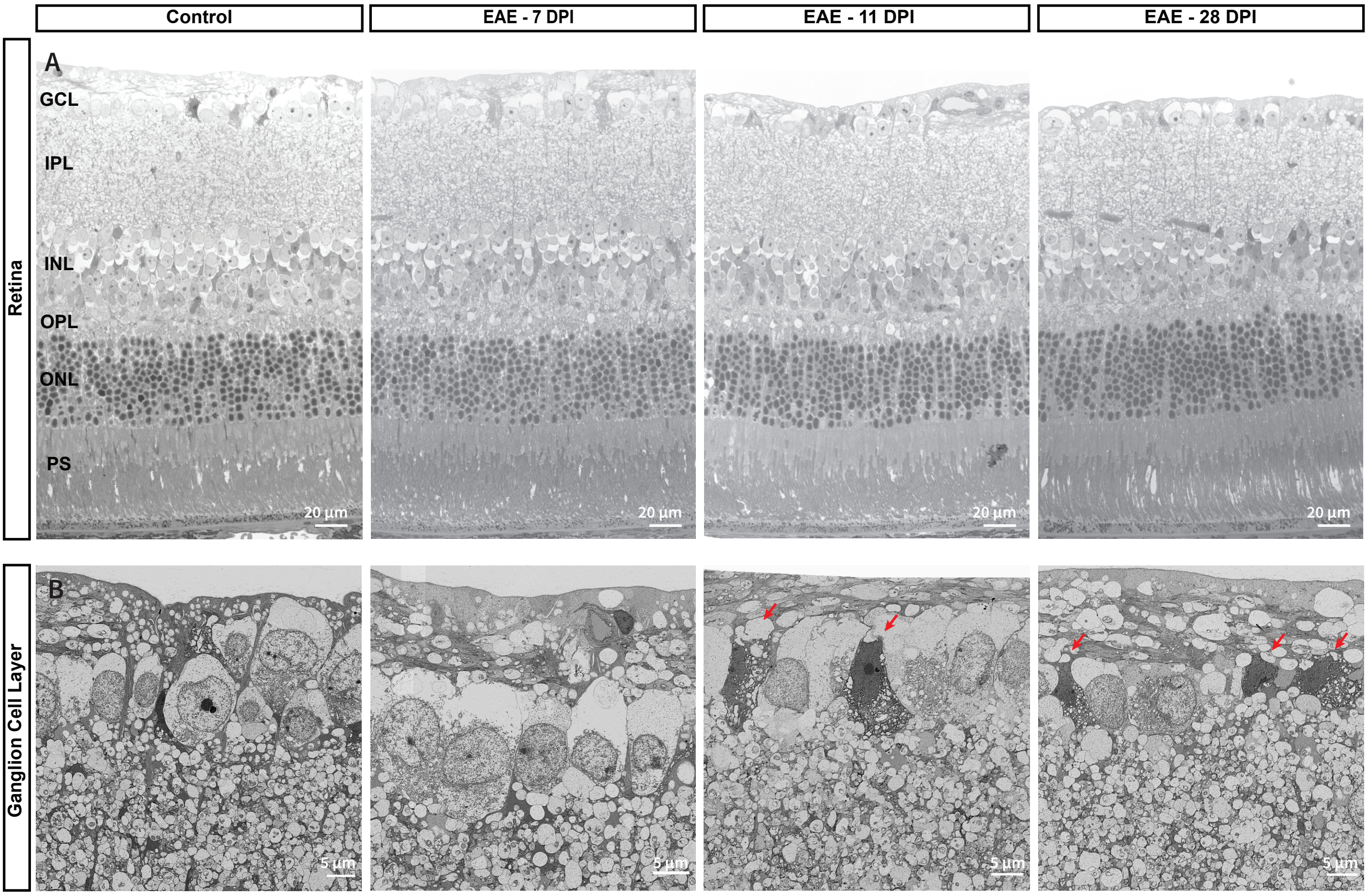

Additional File 2. Retinal degeneration was observed in the ganglion cell layer in EAE mice.
(A) No notable gross changes were observed in EAE retinal light micrographs. (B) Scanning electron micrographs of retinal sections in healthy and EAE mice. In the retina, degeneration of cell bodies in the ganglion cell layer was observed at 11 and 28 dpi (red arrows).

EAE: experimental autoimmune encephalomyelitis, GCL: ganglion cell layer, IPL: inner plexiform layer, INL: inner nuclear layer, OPL: outer plexiform layer, ONL: outer nuclear layer, PS: photoreceptor segment.

Retinal pathology in experimental optic neuritis is characterized by retrograde degeneration and gliosis.
Praveena Manogaran*[1,2], Marijana Samardzija, Anaïs Nura Schad, Carla Andrea Wicki, Christine Walker-Egger, Markus Rudin, Christian Grimm, Sven Schippling.
[1]Department of Information Technology and Electrical Engineering, Swiss Federal Institute of Technology, Zurich, Switzerland
[2]Neuroimmunology and Multiple Sclerosis Research, Clinic for Neurology, University Hospital Zurich and University of Zurich, Zurich, Switzerland
*pmanogar@student.ethz.ch
